# Histone methylation regulates reproductive diapause in *Drosophila melanogaster*

**DOI:** 10.1371/journal.pgen.1010906

**Published:** 2023-09-13

**Authors:** Abigail DiVito Evans, Regina A. Fairbanks, Paul Schmidt, Mia T. Levine

**Affiliations:** 1 Department of Biology, School of Arts and Sciences, University of Pennsylvania, Philadelphia, Pennsylvania, United States of America; 2 Epigenetics Institute, University of Pennsylvania, Philadelphia, Pennsylvania, United States of America; Instituto Leloir, ARGENTINA

## Abstract

Fluctuating environments threaten fertility and viability. To better match the immediate, local environment, many organisms adopt alternative phenotypic states, a phenomenon called “phenotypic plasticity.” Natural populations that predictably encounter fluctuating environments tend to be more plastic than conspecific populations that encounter a constant environment, suggesting that phenotypic plasticity can be adaptive. Despite pervasive evidence of such “adaptive phenotypic plasticity,” gene regulatory mechanisms underlying plasticity remains poorly understood. Here we test the hypothesis that environment-dependent phenotypic plasticity is mediated by epigenetic factors. To test this hypothesis, we exploit the adaptive reproductive arrest of *Drosophila melanogaster* females, called diapause. Using an inbred line from a natural population with high diapause plasticity, we demonstrate that diapause is determined epigenetically: only a subset of genetically identical individuals enter diapause and this diapause plasticity is epigenetically transmitted for at least three generations. Upon screening a suite of epigenetic marks, we discovered that the active histone marks H3K4me3 and H3K36me1 are depleted in diapausing ovaries. Using ovary-specific knockdown of histone mark writers and erasers, we demonstrate that H3K4me3 and H3K36me1 depletion promotes diapause. Given that diapause is highly polygenic, that is, distinct suites of alleles mediate diapause plasticity across distinct genotypes, we also investigated the potential for genetic variation in diapause-determining epigenetic marks. Specifically, we asked if these histone marks were similarly depleted in diapause of a genotypically distinct line. We found evidence of divergence in both the gene expression program and histone mark abundance. This study reveals chromatin determinants of phenotypic plasticity and suggests that these determinants may be genotype-dependent, offering new insight into how organisms may exploit and evolve epigenetic mechanisms to persist in fluctuating environments.

## Author summary

Fluctuating environments pose significant challenges to developing organisms. To better match the immediate, local environment, many organisms follow distinct developmental trajectories in distinct environments, a phenomenon called “phenotypic plasticity.” Distinct developmental trajectories are associated with genome-wide changes in gene expression; however, we have a limited understanding of how these gene expression changes are regulated genome-wide. To address this gap, we exploit the model fruit fly, *Drosophila melanogaster*, which reversibly arrests reproduction in response to cues of oncoming winter. Here we show that epigenetic mechanisms mediate this environment-dependent reproductive plasticity. We also find that these epigenetic mechanisms are genetically variable across distinct genotypes. These new discoveries highlight the importance of reversible epigenetic changes in mediating phenotypic plasticity while establishing a new, genetically tractable model for studying the mechanisms and evolution of phenotypic plasticity. Understanding how organisms persist, or fail to persist, in response to fluctuating environmental conditions is increasingly urgent in face of climate change.

## Introduction

Fluctuating environments threaten survival and reproduction in natural populations. The evolution of environment-dependent, phenotypic plasticity promotes the development of alternative phenotypes that better match the immediate, local environment. For example, decreased daylength linked to seasonal snow cover triggers a coat color change from brown to white in the boreal snowshoe hare (*Lepus americanus*). Increased daylength linked to melting snow triggers redevelopment of a brown coat [1]. Similarly, limited resource availability triggers *Caenorhabditis elegans* juveniles to enter physiological arrest. Once resource availability improves, the juveniles resume development into adults [2,3]. Across a species’ range, variation in the degree of environmental fluctuation may select for different degrees of phenotypic plasticity. Despite the clear relevance of such adaptive phenotypic plasticity in organismal and population responses to a changing climate, the molecular mechanisms that determine environment-induced plasticity are poorly understood [4–8].

Alternative plastic phenotypes are determined by coordinated up- and down- regulation of large swaths of the genome in response to changes in environmental conditions [reviewed in [5,6]]. The molecular mechanisms that regulate these alternative gene expression programs associated with phenotypic plasticity are largely unknown. In contrast, the gene regulatory mechanisms of cell fate plasticity are well-established. Epigenetic mechanisms such as DNA packaging into alternative “chromatin states” regulate cell fate by determining distinct gene expression programs and, ultimately, distinct cellular identities [[9–12] reviewed in [13–15]]. These alternative chromatin states include differential chemical modifications to either the DNA or the histone proteins that make up the nucleosome around which DNA wraps. The addition and removal of acetyl and methyl groups from histone tails can alter the transcriptional state of the underlying DNA and promote distinct cell fates in response to intrinsic developmental cues [[16,17], reviewed in [18,19]]. Intriguingly, extrinsic environmental cues can also alter DNA packaging into chromatin [20–26]. Drought, temperature, salinity, and exposure to toxins alter the genome-wide distribution and abundance of acetyl and methyl groups on histone tails [reviewed in [27,28]]. The observation that chromatin state is both environment-sensitive and a key determinant of cell fate during development raises the possibility that chromatin may mediate environment-sensitive phenotypic plasticity [29].

Consistent with this possibility, a handful of studies have established causal links between chromatin and phenotypic plasticity [29–34]. Three of these studies probe chromatin-based regulation of reversible developmental arrest [32–34]. To escape unfavorable environmental conditions, some organisms have evolved a state of dormancy in which development is suspended and senescence is slowed [35]. Dormancy can occur at any developmental stage, from embryo to adult. In juvenile dormancy, a paused transition between developmental stages results in dramatic lifespan extensions. In adult dormancy, both somatic lifespan and reproductive lifespan are extended [36]. While several groundbreaking studies have identified chromatin-based regulation of juvenile dormancy [32–34], the chromatin determinants of adult dormancy, and specifically the adaptive preservation of reproductive potential at this life stage, have not yet been explored. Moreover, we know virtually nothing about natural genetic variation in the epigenetic mechanisms that promote and constrain phenotypic plasticity [37].

To investigate chromatin-based regulation of adaptive reproductive preservation in dormancy, we exploit the tractable model system, *Drosophila melanogaster*. *D*. *melanogaster* enters a form of adult dormancy, called diapause, in response to the cold temperatures and short days of oncoming winter [as defined in [38–41], but see [42]]. This reversible arrest is adaptive; indeed, in the absence of diapause, females fail to survive the winter [36,43,44]. *Drosophila* female diapause is characterized by extensive physiological changes that result in increased lipid storage, increased stress tolerance, increased lifespan extension, and suspended egg production. Suspended egg production is associated with global changes to the ovary transcriptome [43,45–49] and results in retention of nearly full reproductive potential following diapause [36,40,43,50]. Global changes to the ovary transcriptome under diapause implicates chromatin regulation, making diapause an ideal model to study the epigenetic determinants of reproductive dormancy.

*D*. *melanogaster* diapause is also an ideal model for investigating the evolution of gene regulatory mechanisms that mediate plasticity. Diapause plasticity is highly polygenic such that genetically distinct individuals have only partially overlapping suites of alleles that promote diapause [51]. Diapause plasticity also varies adaptively [47,52,53]. In populations from high latitudes with extreme winters, a higher proportion of females enter diapause under simulated winter conditions than females from low latitudes with mild winters [47,52,53]. Similarly, a higher proportion of females enter diapause in populations collected immediately following winter than those collected in the late summer [53]. This spatial- and temporal- variation in diapause plasticity, along with the observation that diapause plasticity is highly polygenic in *D*. *melanogaster*, makes this system ideal for probing how epigenetic determinants of plasticity vary across distinct genotypes.

Here we identify two epigenetic factors that regulate reproductive diapause through a mechanism distinct from those previously identified in juvenile diapause [32–34]. We also show that these epigenetic marks may vary across distinct genotypes. These data provide new insight into how organisms exploit epigenetic mechanisms to persist in fluctuating environments, and how genetic variation may shape these epigenetic mechanisms.

## Results

### Establishing a system to study epigenetic regulation of reproductive plasticity

To study the epigenetic determinants of reproductive lifespan extension under diapause, we established a system wherein epigenetic regulation, including chromatin-mediated gene regulation, could be isolated from the often-confounding effects of genotype, environment, and tissue heterogeneity. *D*. *melanogaster* diapause emerged as a compelling candidate system. This system allows us to control for genetic variation, to evaluate alternative developmental states in the same environment, and to ensure tissue homogeneity across alternative reproductive states.

**Fig 1:**
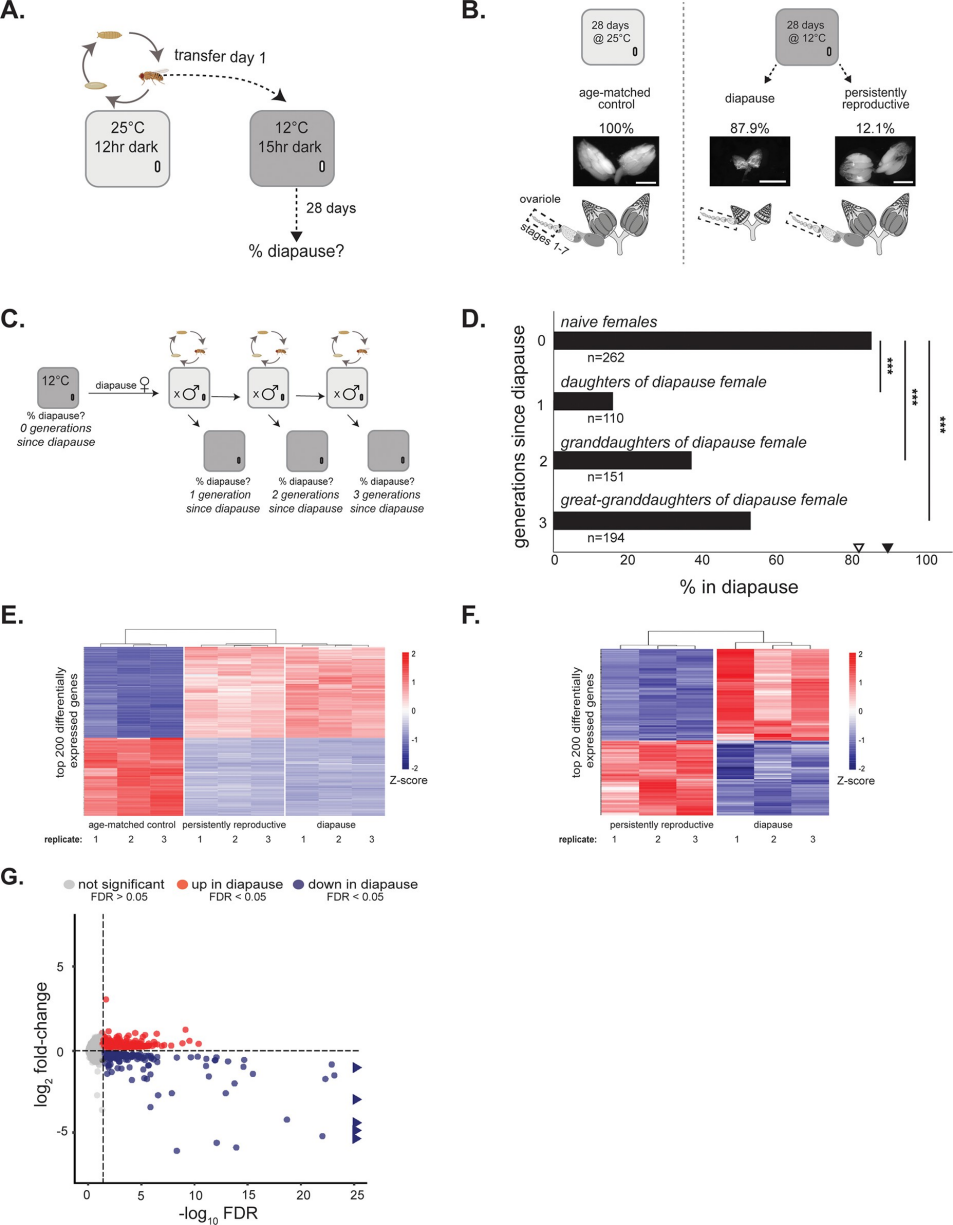
Establishing a system to study epigenetic regulation of adaptive phenotypic plasticity. (A) Diagram of diapause assay design. Flies are reared at 25°C under a 12-hour dark/light cycle in an incubator (light gray). Females are then transferred to an incubator set to 12°C under a 15-hour dark, 9-hour light cycle (dark gray, simulated winter conditions). Females are maintained in simulated winter conditions for 28 days before ovaries are assessed for diapause (% of females with arrested ovaries, i.e., “diapause plasticity”). (B) Degree of plasticity in age-matched control at 25°C (left) and diapause and persistently reproductive at 12°C (right). Ovaries from age-matched control females, diapause females, and persistently reproductive females and cartoons (below) representing the ovaries with a separated single ovariole, the basic unit of egg production in the *Drosophila* ovary. Dotted box indicates stages 1–7 used for all RNA and protein assays (note that the arrested ovary has only stages 1–7, created using Biorender.com). Scale bars = 0.5 mm (C) Diagram of transgenerational assay design. After maintenance under simulated winter conditions for 28 days (dark gray incubator, see above), females are assayed for diapause using a non-destructive method (see Methods). The females previously in diapause (“naïve females”) are then crossed to males at 25°C. Virgin females from this cross (“daughters of diapause female”) are placed either into a 12°C incubator to assess diapause plasticity or into a vial with males at 25°C to generate the granddaughters of diapause females. This process is repeated with these granddaughters and the great-granddaughters of diapause females. (D) Diapause plasticity of daughters, granddaughters, and great-granddaughters of females who underwent diapause. χ^2^, *** p<0.001, n = total number of females assayed. The filled triangle represents the plasticity of naïve females collected in parallel to the first half of the experiment and the empty triangle represents the plasticity of naïve females collected in parallel to the second half of the experiment (S1 Table) (E) Heatmap of the top 200 differentially expressed genes (by false discovery rate, “FDR”) between age-matched control, diapausing, and persistently reproductive ovaries. Blue-red gradient depicts the Z-score of each gene. Red corresponds to upregulated genes and blue corresponds to downregulated genes. (F) Heatmap of top 200 differentially expressed genes (by FDR) between diapause and persistently reproductive ovaries. Blue-red gradient depicts the Z-score of each gene. Red corresponds to upregulated genes and blue corresponds to downregulated genes. (G) Volcano plot showing differential gene expression across diapausing and persistently reproductive ovaries. Triangles represent genes with -log_10_ FDR > 25. Cartoons created with BioRender.com.

To control for genotypic effects on epigenetic regulation, we inbred an isofemale line from a temperate North American population (collected in Media, Pennsylvania) by brother-sister mating for 10 generations. Under simulated winter conditions (Fig 1A), 87.9% of inbred females enter diapause (Fig 1B). Importantly, the incidence of diapause in this inbred line does not differ from the isofemale line from which it was derived (χ^2^ test, p = 0.49), suggesting that residual, segregating genetic variation alone does not account for the observed degree of plasticity. Incomplete diapause penetrance, where most females arrest but some (12.1%) remain persistently reproductive upon exposure to simulated winter conditions, allows us to control for environment in addition to genotype: we can compare the chromatin state of inbred diapausing and persistently reproductive individuals in the same environment. Finally, to control for tissue heterogeneity in all experiments that compared arrested and persistently reproductive ovaries, we isolated ovary stages 1–7 (see Methods). Stages 1–7 are those represented in diapause (Fig 1B). This careful exclusion of development beyond stage 7 allowed us to control for cell type composition between arrested and persistently reproductive ovaries.

The observation of environment-induced alternative phenotypic states across individuals of a single genotype implicates epigenetic regulation. Another hallmark of epigenetic regulation is transgenerational transmission of parental environmental conditions to offspring [54–57]. To probe the possibility that parental diapause is transmitted to offspring, we assayed diapause plasticity (the proportion of females that enter diapause) of daughters, granddaughters, and great-granddaughters of inbred females that had undergone diapause (Fig 1C). We first subjected a cohort of females to winter conditions (12°C, Fig 1C “naïve females”). We allowed the subset of females that had entered diapause to mate and reproduce at 25°C. We assayed one cohort of their daughters for diapause plasticity (Fig 1C “daughters”, one generation since diapause) and allowed a second cohort to mate and reproduce at 25°C. We repeated this process until the great-granddaughter generation (Fig 1C “great granddaughters”, three generations since diapause). We note that, under this protocol, the *propagated* daughters, granddaughters, and great-granddaughters are never exposed to winter conditions. A distinct subset of these progeny was sampled every generation to determine diapause propensity and then discarded. These experiments revealed that diapause entry in mothers reduces the proportion of daughters in diapause (χ^2^, p<2.2x10^-16^), granddaughters in diapause (χ^2^, p<2.2x10^-16^), and great-granddaughters in diapause (χ^2^, p = 4.6x10^-8^, Fig 1D). This reduction in diapause plasticity in the daughters of mothers who had experienced diapause is typically observed when an unfavorable maternal environment (e.g., winter) predicts a favorable progeny environment [e.g., spring [58,59], see Discussion]. Moreover, the observed transgenerational effect decreases with each generation removed from the initial incidence of diapause. The dilution of the transgenerational effect suggests dilution of an epigenetic signal through generations [[60], reviewed in [61]]. Such transgenerational effects in an inbred line further implicate a role for epigenetic regulation of diapause plasticity.

A classic readout of epigenetically regulated, alternative phenotypic fates is alternative gene expression programs across genetically identical individuals from identical environments [62]. To profile gene expression across the two reproductive fates, we performed RNA-seq on both arrested and persistently reproductive ovaries from females maintained under simulated winter conditions for 28 days (see Methods). We also performed RNA-seq on ovaries from age-matched control females maintained at 25°C for 28 days. We prepared RNA from exclusively stages 1–7 in both arrested and reproductive ovaries to ensure tissue homogeneity between samples (Fig 1B).

RNA-seq revealed distinct gene expression profiles of age-matched control ovaries (25°C), arrested ovaries (12°C), and persistently reproductive ovaries (12°C). Consistent with the well-documented, pervasive effects of temperature alone on gene expression [63,64], the 200 most differentially expressed genes (by false discovery rate, “FDR”) are differentially expressed between age-matched control ovaries at 25°C and ovaries at 12°C (Figs 1E and S1A and S2 Table); however, within the 12°C treatment, diapausing and persistently reproductive ovaries have distinct gene expression programs (Figs 1F and S1B and S3 Table). Slightly more genes are down-regulated than up-regulated in diapausing compared to persistently reproductive ovaries (411 and 352, respectively), and many more down-regulated genes have log_2_-fold change greater than two (Fig 1G). Nevertheless, the significant upregulation of hundreds of genes in diapause suggests that *D*. *melanogaster* diapause is not simply a generalized shut-down of gene expression but instead an actively regulated state [see also [49]]. This differential gene expression between diapause and persistently reproductive ovaries at 12°C, combined with the transgenerational effect of diapause, suggests that epigenetic factors mediate reproductive arrest in the ovary.

### Epigenetic marks H3K4me3 and H3K36me1 regulate diapause plasticity

**Fig 2:**
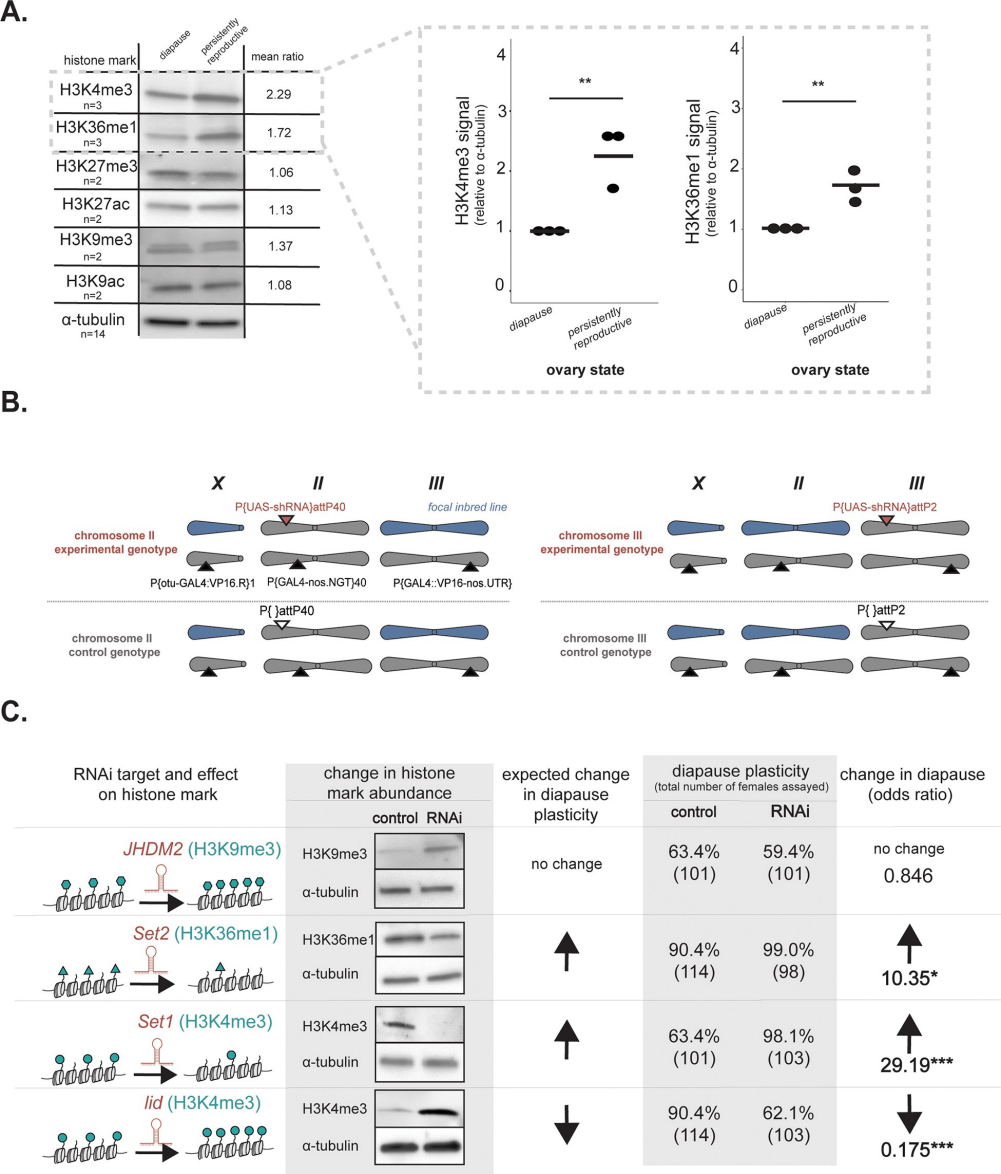
H3K4me3 and H3K36me1 regulate diapause plasticity. (A) Representative western blots showing histone mark abundance in diapausing and persistently reproductive ovaries. Quantification of H3K4me3 and H3K36me1 signal relative to α-tubulin loading control for three biological replicates (right). t-test, **p < 0.01, n = number of replicate blots, mean ratio = persistant reproductive:diapause (B) Genotypes of experimental and control lines in histone mark manipulation experiment. Blue chromosomes represent chromosomes from the focal inbred line, while gray chromosomes represent chromosomes that have either the MTD-Gal4 drivers integrated (depicted by black triangles) or the RNAi construct (TRiP) integrated. Experimental genotypes (above dotted line) encode a construct with a UAS promoter that drives a small hairpin RNA (“P{UAS-shRNA}”). These constructs are inserted into a chromosome-specific attP site (pink triangle). Control genotype attP sites lack the inserted construct (white triangle). (C) Strategy, expected results, and observed results from histone mark writer or eraser knockdown, including western blots validating histone mark depletion or enrichment and the change in likelihood of diapause given writer or eraser knockdown, FET, * p < 0.05, *** p < 0.001. Note that we ruled out the possibility that activation of RNAi alone impacts diapause (see Methods) and the possibility that diapause-independent effects of knockdown of *Set1* and *Set2* on ovary development confounded these results (see Methods and S3B Fig).

Epigenetic regulation depends in part on chromatin modifications [reviewed in [65]], including post-translational histone modifications (“histone marks”), that alter the transcriptional activity of the underlying DNA [reviewed in [66]]. To identify histone marks associated with diapause plasticity, we prepared lysate from arrested ovaries and persistently reproductive ovaries (stages 1–7 only) and screened six, highly abundant histone H3 modifications [67]. Given the strong downregulation of many genes in diapausing ovaries (Fig 1G), we predicted either an excess of repressive marks or the depletion of active marks. The screen revealed that repressive marks H3K27me3 and H3K9me3, as well as active marks H3K27ac and H3K9ac, did not differ in abundance across diapausing and persistently reproductive ovaries (Figs 2A and S2 and S4 Table). In contrast, active marks H3K4me3 and H3K36me1 were depleted in diapause (t-test, p<0.01, Figs 2A and S2).

The depletion of active marks in diapausing ovaries could be a byproduct of the strong downregulation of many genes in diapause or could reflect a causal role of histone marks in determining diapause plasticity. To test the prediction that the abundance of these marks in the ovary affect reproductive plasticity, we manipulated histone mark abundance. Using the Gal4/UAS system (see Methods), we expressed in the ovary short hairpin RNAs (shRNAs) that knock down transcripts of enzymes that deposit or remove these histone marks (“writers” and “erasers,” respectively, Fig 2B and 2C). Given that diapause plasticity is well-known to vary across genotypes [46,48,68,69], we strictly controlled the genetic background of the experimental and control flies. To generate the experimental lines, we introduced chromosomes carrying the focal shRNA construct, integrated into an attP landing site, into the inbred line from Pennsylvania described above (Fig 2B). To generate the control lines, we introduced chromosomes that have the same attP landing site, but *lack* the shRNA construct, into the same inbred line (Fig 2B). To establish that RNAi alone does not affect diapause, we confirmed that RNAi against mCherry behaved similarly to our controls lines that lack an RNAi construct (see Methods). We crossed these experimental and control lines to a driver line that directs expression of the shRNA in the ovary.

We manipulated the abundance of three histone marks in the ovary and assayed diapause plasticity (the proportion of females with arrested ovaries) using these rigorously controlled genotypes. First, we manipulated a “control” histone mark, H3K9me3, which did not vary between diapausing and reproductive ovaries (Figs 2A and S2). Specifically, we knocked down *JHDM2* (S3A Fig), an enzyme that demethylates H3K9. As expected, *JHDM2* knockdown elevated H3K9me3 but had no effect on diapause plasticity (Fig 2C, odds ratio = 0.846, Fisher’s Exact Test, FET, p>0.05). Next, we manipulated H3K36me1 and H3K4me3, two histone marks depleted in arrested ovaries (Fig 2A). We predicted that experimental depletion of these marks would increase diapause plasticity, while experimental enrichment would decrease diapause plasticity. This is exactly what we observed. To deplete H3K36me1, we knocked down *Set2*, which encodes an enzyme that methylates H3K36 (Figs 2C and S3A). Indeed, H3K36me1 depletion increased diapause plasticity (Fig 2C, odds ratio = 10.35, FET, p<0.05). Similarly, we depleted H3K4me3 by knocking down *Set1*, which encodes an enzyme that methylates H3K4 (Figs 2C and S3A) and again observed increased diapause plasticity (Fig 2C, odds ratio = 29.19, FET p<0.0001). We then experimentally *enriched* H3K4me3 by knocking down *lid*, which encodes an enzyme that removes H3K4 methylation (Figs 2C and S3A). As predicted, this opposing manipulation decreased diapause plasticity (Fig 2C, odds ratio = 0.175, FET, p<0.0001). Importantly, observing this opposing effect of decreased diapause blunted our concern that active mark depletion simply blocks ovary development beyond stage 7. Furthermore, our observation of many persistently reproductive ovaries upon depletion of H3K36me1 and of H3K4me3 in the context of the diapause plasticity-increasing transgenerational effect also rejected the possibility that compromised ovary development confounds our results (S3B Fig, see Methods). Together, these data suggest that H3K36me1 and H3K4me3 depletion, but not H3K9me3 elevation, promotes diapause plasticity.

### Diapause-associated chromatin state and gene expression are genotype-specific

**Fig 3:**
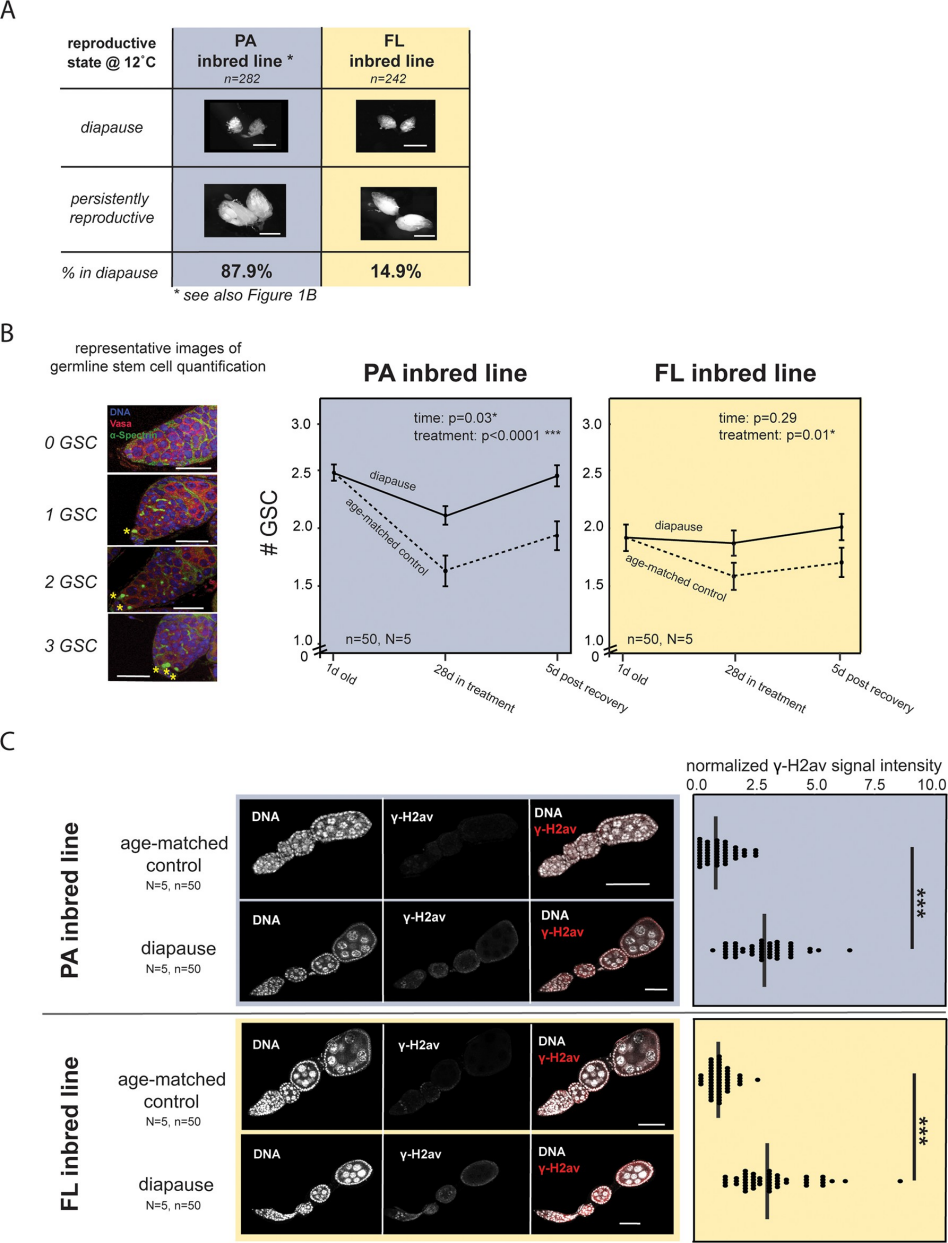
Assessing the diagnostic features of diapause in a low plasticity line. (A) Representative images of diapausing and persistently reproductive ovaries of high plasticity (PA, blue) and low plasticity (FL, yellow) inbred lines as well as the degree of plasticity (note: PA % diapause was reported previously in Fig 1B). Scale bar = 0.5 mm, n = total number of females assayed. (B) Representative images of germaria with 0, 1, 2, and 3 germline stem cells (“GSC,” designated by *, scale bar = 25μm) stained with DAPI (blue), anti-Vasa (red) and anti-α-Spectrin (green). Average number of germline stem cells at one day old, after 28 days of treatment (25°C or diapause), and after five days post-treatment at 25°C for diapausing and age-matched control ovaries of PA and FL females (d =“day”, 2-way ANOVA with fixed effects = timepoint, treatment, error bars = SEM, N = number of assayed ovaries, n = number of assayed ovarioles). (C) γ-H2av signal in age-matched control and diapausing ovaries of PA and FL females and quantification. Mann-Whitney U test, *** p<0.001, scale bar = 50μm, N = number of assayed ovaries, n = number of assayed ovarioles.

Diapause plasticity in *D*. *melanogaster* is a highly polygenic trait that varies both geographically and seasonally, as described above [47,51–53]. Because diapause is determined by variation at hundreds of genes, geographically distinct populations share only partially overlapping alleles that promote (or constrain) diapause plasticity. This distinct genetic architecture predicts distinct transcriptional programs across natural populations and raises the possibility that distinct epigenetic mechanisms contribute to diapause plasticity across distinct genotypes. To explore these predictions, we inbred an additional, geographically distinct line collected from Florida. This inbred line has low diapause plasticity (14.9% diapause, Fig 3A). Henceforth, we refer to the original inbred line described above as “PA”, and the Florida inbred line as “FL”.

Given that the FL line is largely insensitive to the simulated winter conditions, we first determined whether diapause in the FL line is a *bona fide* alternative developmental state or instead is a generalized stress response to simulated winter conditions [see [70]]. The *D*. *melanogaster* female’s generalized stress response to unfavorable environmental conditions, such as starvation or predator exposure, manifests superficially as arrested ovary development [71–74]. However, generalized stress response in the ovaries is both cell biologically and functionally distinct from diapause [50]. Previous studies have demonstrated that diapause preserves fertility and germline stem cell number compared to age-matched controls, while stress does not [50]. Diapausing ovaries also accumulate the double-strand break marker, γ-H2av, due to the persistence of egg chambers in extended arrest [50]. We used these cell biological markers of diapause to confirm that the arrested ovaries in the FL line are, in fact, undergoing diapause rather than a generalized stress response. We found that, despite low responsiveness to simulated winter conditions, the FL line, like the PA line, preserves germline stem cell number both during diapause and five days after diapause recovery compared to age-matched controls (2-way ANOVA with fixed effects, PA: time p = 0.03, treatment p = 0.0001, LP: time p = 0.29, treatment p = 0.01, Fig 3B and S5 Table). Furthermore, FL and PA diapausing ovaries are similarly enriched for γ-H2av compared to age-matched controls (Mann-Whitney U test, *** p<0.001 in both genotypes, Fig 3C and S6 Table). Diapause also preserves fertility in the FL line compared to age-matched controls (S4 Fig, t-test, PA p<0.001, FL p<0.01, S7 Table). These data suggest that the FL line enters a true diapause state in response to simulated winter conditions.

**Fig 4:**
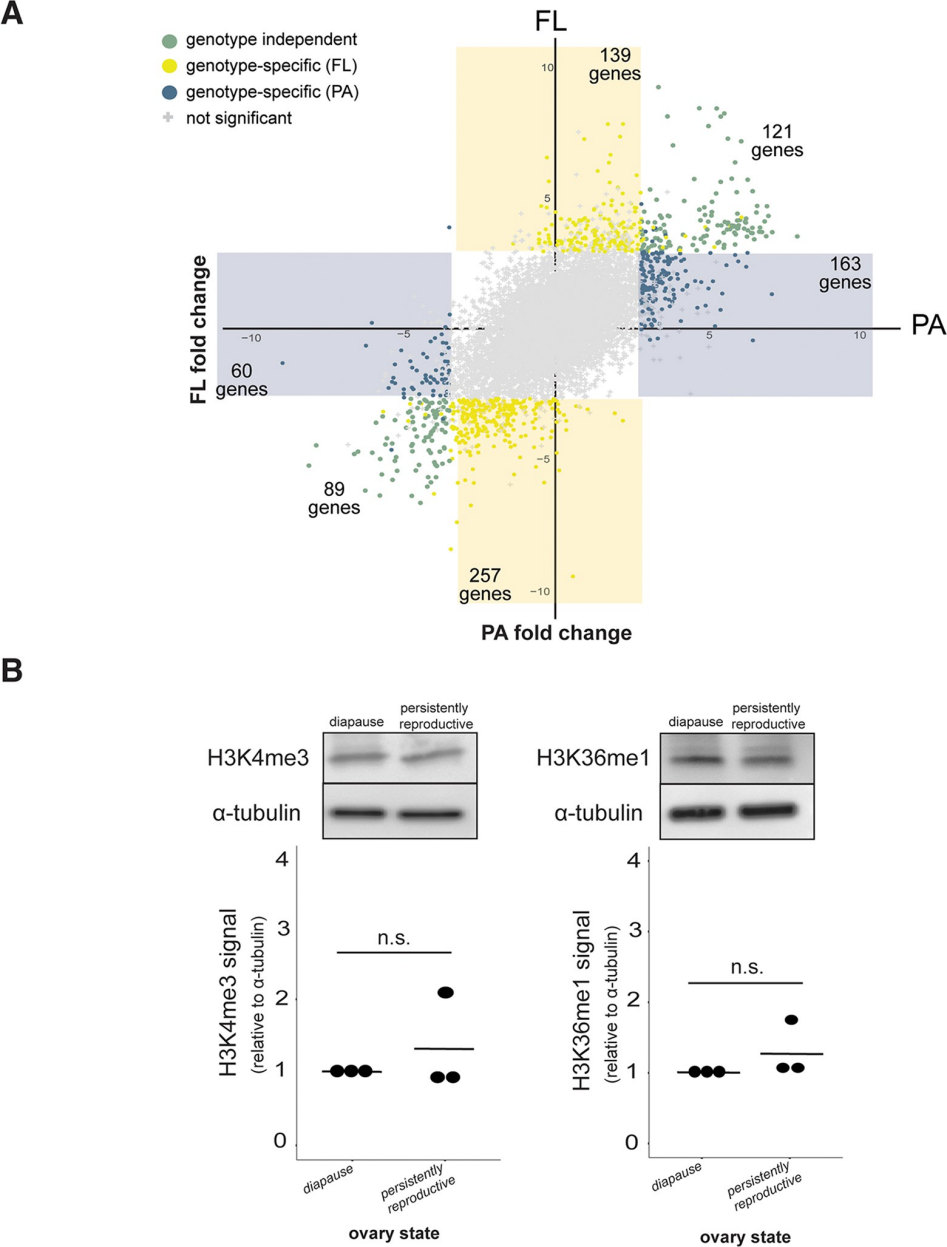
Diapause-associated gene expression and diapause-associated chromatin state are genotype-specific. (A) Differential gene expression across diapausing and persistently reproductive ovaries in PA and FL genotypes. Gray points represent genes that are not significantly differentially expressed in either genotype. Green points represent genes that are differentially expressed in both genotypes (FDR < 0.05). Blue points represent genes that are differentially expressed in the PA genotype only (FDR < 0.05). Yellow points represent genes that are differentially expressed in the FL genotype only (FDR < 0.05). (B) Representative western blots showing the abundance of H3K4me3 and H3K36me1 in diapausing and persistently reproductive ovaries from the FL inbred genotype females (above). Quantifications of H3K4me3 and H3K36me1 signal relative to α-tubulin loading control for three replicates (below). t-test, n.s. = p>0.05, (compare to Fig 2A).

To investigate whether the epigenetic mechanisms mediating plasticity are distinct across diverged genotypes, we first asked if genetic variation in diapause plasticity manifests as transcriptional variation. We conducted RNA-seq on diapausing and persistently reproductive ovaries from the FL line and compared the differentially expressed genes to those of the PA line (Figs 1E and S5 and S2–S3, S8–S10 Tables). To isolate the subset of genes that are up- and down-regulated specifically in diapause, we normalized the list of genes that were differentially expressed between diapausing and persistently reproductive ovaries (both at 12°C) to gene expression of age-matched control ovaries (25°C). Specifically, we included in downstream analyses only those genes that were differentially expressed between diapausing and persistently reproductive ovaries *and* between diapausing and age-matched control ovaries in a given genotype, removing genotype-restricted expression specific to the persistently reproductive ovaries. We compared this reduced list of diapause-specific genes across the two genotypes (433 in PA, 606 in LP) and determined which genes were differentially expressed in both PA and FL (“genotype-independent”), differentially expressed only in the PA line (“PA-specific”), or differentially expressed only in the FL line (“FL-specific”). While many genes that are up- or down- regulated in diapause are shared across the PA and FL lines (201 genes, Fig 4A), most differentially expressed genes are genotype-dependent (611 genes, Fig 4A). These results suggest that PA and FL diapause plasticity are associated with only partially overlapping transcriptional programs, which is consistent with the known polygenic basis of diapause plasticity,

We predicted that the genes up- or down-regulated in diapausing ovaries in only one genotype (“genotype-dependent”) may regulate pathways that promote reproductive arrest common to both genotypes. To test this prediction, we first conducted pathway analysis on PA-specific, FL-specific, and genotype-independent genes that were significantly differentially expressed (FDR <0.05, greater than five genes per pathway). We then assessed overlap across these three classes. Consistent with our prediction, we found evidence that genotype-dependent gene expression in PA and FL diapause converges on common biological processes. For example, two metabolic pathways involved in ATP synthesis (“The citric acid (TCA) cycle and respiratory electron transport” and “Respiratory electron transport, ATP synthesis by chemiosmotic coupling, and heat production by uncoupling proteins”) are enriched in both PA-specific and FL-specific genes up-regulated in diapause (Table 1, highlighted in green). Moreover, twelve pathways are enriched for *both* genotype-independent genes and genotype-*dependent* genes upregulated in diapause (Table 1, highlighted in gray). This finding suggests that some pathways are utilized by both genotypes via the expression of overlapping genes *and* non-overlapping genes. There were no significant pathways overrepresented for genes downregulated in PA-dependent genes and only a single pathway overrepresented for genotype-independent downregulated genes; consequently, common pathways could not be detected for down-regulated genes. These results suggest that diapause in both genotypes depends on the activation of common pathways despite pervasive genotype-dependent gene expression.

**Table 1:** Pathway enrichment for genes up-regulated in diapause. Pathways in gray cells are significantly enriched in all categories (genotype-independent, PA-specific, and FL-specific genes). Pathways in green cells are significantly enriched in both PA-specific and FL-specific genes. Pathways in blue cells are significantly enriched in both genotype-independent and PA-specific genes. Pathways in white cells are significantly enriched in only one genotype. No pathways are significant in both genotype-independent and FL-specific genes.

Pathway (pathway identifier)	genotype-independent FDR (gene count)	PA-specific FDR (gene count)	FL-specific FDR (gene count)
GTP hydrolysis and joining of the 60S ribosomal subunit (R-DME-72706)	4.08E-102 (65)	4.21E-03 (6)	4.20E-02 (5)
SRP-dependent cotranslational protein targeting to membrane (R-DME-1799339)	3.82E-101 (65)	5.49E-03 (6)	4.99E-02 (5)
Nonsense Mediated Decay (NMD) independent of the Exon Junction Complex (EJC) (R-DME-975956)	1.38E-100 (65)	6.25E-03 (6)	1.68E-02 (6)
Formation of a pool of free 40S subunits (R-DME-72689)	1.58E-99 (65)	3.93E-06 (10)	3.12E-03 (7)
L13a-mediated translational silencing of Ceruloplasmin expression (R-DME-156827)	1.87E-99 (66)	4.95E-06 (10)	1.43E-03 (8)
Eukaryotic Translation Initiation (R-DME-72613)	4.93E-98 (66)	6.47E-06 (10)	1.46E-03 (8)
Cap-dependent Translation Initiation (R-DME-72737)	5.54E-98 (66)	7.76E-06 (10)	1.82E-03 (8)
Nonsense Mediated Decay (NMD) enhanced by the Exon Junction Complex (EJC) (R-DME-975957)	5.61E-98 (65)	1.17E-02 (6)	2.24E-02 (6)
Nonsense-Mediated Decay (NMD) (R-DME-927802)	6.55E-98 (65)	1.23E-02 (6)	2.41E-02 (6)
Translation (R-DME-72766)	1.99E-84 (67)	1.98E-05 (12)	1.43E-03 (11)
Metabolism of RNA (R-DME-8953854)	1.26E-70 (71)	1.85E-03 (14)	1.84E-03 (15)
Metabolism of proteins (R-DME-392499)	1.62E-47 (71)	5.59E-05 (25)	1.76E-02 (21)
The citric acid (TCA) cycle and respiratory electron transport (R-DME-1428517)	n.s.	8.59E-04 (9)	1.49E-02 (8)
Respiratory electron transport, ATP synthesis by chemiosmotic coupling, and heat production by uncoupling proteins. (R-DME-163200)	n.s.	2.92E-03 (7)	2.88E-02 (6)
Ribosomal scanning and start codon recognition (R-DME-72702)	1.08E-38 (29)	5.60E-06 (8)	n.s.
Translation initiation complex formation (R-DME-72649)	1.52E-38 (29)	5.56E-06 (8)	n.s.
Activation of the mRNA upon binding of the cap-binding complex and eIFs, and subsequent binding to 43S (R-DME-72662)	2.13E-38 (29)	5.60E-06 (8)	n.s.
Formation of the ternary complex, and subsequently, the 43S complex (R-DME-72695)	3.94E-38 (28)	5.79E-06 (8)	n.s.
rRNA processing in the nucleus and cytosol (R-DME-8868773)	2.66E-20 (20)	3.76E-02 (5)	n.s.
rRNA processing (R-DME-72312)	2.80E-20 (20)	3.90E-02 (5)	n.s.
Major pathway of rRNA processing in the nucleolus and cytosol (R-DME-6791226)	2.97E-20 (20)	4.05E-02 (5)	n.s.
Metabolism (R-DME-1430728)	n.s.	1.16E-05 (7)	n.s.
Signaling by Nuclear Receptors (R-DME-9006931)	n.s.	3.92E-02 (6)	n.s.
Metabolism of amino acids and derivatives (R-DME-71291)	n.s.	4.42E-02 (28)	n.s.
mRNA Splicing (R-DME-72172)	n.s.	n.s.	2.91E-02 (7)
mRNA Splicing–Major Pathway (R-DME-72163)	n.s.	n.s.	3.04E-02 (7)

Pervasive genotype-dependent gene expression raises two alternative hypotheses that predict distinct epigenetic regulation of diapause across distinct genotypes. Diapause plasticity in the PA and FL lines could be regulated by distinct epigenetic marks at the distinct sets of genes associated with diapause. Alternatively, the same epigenetic mark, like H3K4me3, could be depleted at distinct sets of genes across the two genotypes. To evaluate these alternative hypotheses, we attempted to use CUT&RUN, a genome-wide chromatin profiling approach that requires minimal sample material [75]. Unfortunately, attempts to profile the minimal tissue from stages 1–7 of the diapausing and persistently reproductive ovaries were unsuccessful. We therefore turned to histone mark abundance to determine whether distinct chromatin states underlie diapause in distinct genotypes. As observed in the PA line, H3K9ac, H3K9me3, H3K27ac and H3K27me3 abundance did not differ between diapausing and persistently reproductive ovaries of the FL line (S2 Fig). However, unlike the H3K4me3 and H3K36me1 depletion that we observed in diapausing ovaries of the PA line, these two marks were invariant across the two reproductive states in the FL line (t-test, p>0.05, Fig 4B). These data are consistent with distinct chromatin states of diapausing ovaries across these two wild-derived genotypes. These data also highlight the idea that downregulation of the genome during diapause is not inevitably associated with loss of a pervasive, active histone mark like H3K4me3.

To determine whether this genetic variation in diapause-dependent histone mark abundance is widespread, we assayed seven additional lines from either PA or FL (S11 Table) for H3K4me3 and H3K36me1 abundance across reproductive states. We found that one additional line shows depleted H3K4me3 and H3K36me1 in diapause (FL-2, t-test, p<0.05), resembling the focal inbred PA line (S6 Fig and S12 Table). We also found that two of the additional lines showed depletion of only H3K4me3 (PA-2, PA-4, t-test, p<0.01). Finally, we found that the remaining three lines showed no depletion of H3K4me3 and H3K36me1 (PA-3, FL-3 and PA-5 S6 Fig). These data suggest that genetic variation in diapause-dependent histone mark abundance is not limited to our two focal lines, consistent with widespread natural variation in plasticity-linked epigenetic marks.

## Discussion

Here we describe a new, tractable system for studying genetic variation in epigenetic regulation of phenotypic plasticity. Importantly, this model system controls for the confounding effects of genetic variation, environment, and tissue heterogeneity on chromatin packaging, allowing us to isolate functional links between chromatin modifications and phenotypic state. We discovered that environment-dependent reproductive arrest in *D*. *melanogaster* is mediated by at least two histone marks, H3K4me3 and H3K36me1. We also found that these epigenetic marks vary genetically–both marks were depleted in diapausing ovaries of some genotypes but were invariant across diapausing and reproductive ovaries in other genotypes. To our knowledge, these data represent the first observation of natural genetic variation in plasticity-associated chromatin marks and raise the possibility of a distinct epigenetic basis of diapause plasticity across distinct genotypes.

Previous studies in non-model or emerging model systems have demonstrated compelling causal links between chromatin and diapause. Two major chromatin silencing pathways emerged from these studies: DNA methylation in parasitoid wasps [34]] and H3K27me3 in the Cotton bollworm moth [33]] and the Turquoise killifish [32]]. *D*. *melanogaster* has minimal DNA methylation [76]; consequently, we focused on histone marks like H3K27me3. Surprisingly, our screen of histone marks revealed no difference in abundance of H3K27me3 between arrested and persistently reproductive ovaries [as was found in bollworm pupa [33]], and we did not detect differential expression of enzymes that write, read, or erase H3K27me3 [as was found in killifish embryos [32]]. These observations implicated a distinct chromatin mechanism regulating *D*. *melanogaster* reproductive diapause. Our focus on the ovary may account for this difference. Previous studies probed paused developmental transitions during juvenile phases, either embryonic or larval, rather than the adult reproductive tissues. Consistently, H3K27me3 is a classic regulator of developmental fate [77]. It is also possible that H3K27me3, along with H3K4me3 and H3K36me1, regulates diapause plasticity but gross H3K27me3 abundance does not vary between reproductive states.

The discovery that H3K4me3 depletion promotes reproductive diapause in the focal PA genotype is reminiscent of earlier studies of somatic aging. From yeast to mammals, aging is associated with a general increase in active chromatin marks like H3K4me3 and an overall increase in transcription [reviewed in [78]]. In *Drosophila*, H3K4me3 depletion extends lifespan [79]. Like these studies of somatic aging, we found not only that H3K4me3 depletion promotes reproductive diapause but also that diapause is associated with a striking down-regulation of gene expression in the PA genotype. These results suggest that the chromatin state of the diapausing PA ovary mirrors that of young somatic tissues. Given that the chromatin determinants of age-dependent reproductive decline [80] are not nearly as well explored as the determinants of somatic aging [reviewed in [78,81]], this system offers a new foothold for understanding how a youthful chromatin state contributes to the preservation of reproductive potential.

Chromatin mediates not only development and aging within a generation but also mediates epigenetic information transfer between generations [reviewed in [82,83]]. Our discovery of distinct epigenetic states between arrested and persistently reproductive ovaries raised the possibility that information about the maternal environment is transferred to the next generation. Consistent with diapause-induced transmission of epigenetic information, we found that diapause entry reduces subsequent diapause plasticity of genetically identical daughters, granddaughters, and great-granddaughters. Transgenerational inhibition of diapause occurs when the maternal environment does not match the offspring environment [84]. This phenomenon has been reported in the flesh fly [*Sacrophaga bullata*,[58]] and in two polyphagous wasps [*Trichogramma telengai* and *Trichogramma principum*,[59]]. For *D*. *melanogaster* females, entering diapause is adaptive only at the onset of winter. The daughters of females that survive the winter are likely to emerge at the start of spring. Being “diapause resistant” allows these daughters to persist as reproductive even if the temperature drops temporarily [84]. The observed transgenerational memory requires a DNA sequence-independent “message” to be passed from parent to offspring through the germline. The identity of this message remains mysterious; however, we speculate that either alternative chromatin packaging or RNA, possibly small RNA(s) [58,85], of diapausing ovaries transmits this heritable epigenetic information. The discovery of transgenerational information transfer from mother to daughter–in a model system–puts forward *D*. *melanogaster* diapause as a powerful new resource for studying intergenerational epigenetic inheritance.

Our discovery of epigenetic regulation of a highly polygenic trait offered us the unique opportunity to explore the effect of genetic variation on epigenetic regulation. This study brings together the historically distinct areas of epigenetic regulation and genetic variation in plasticity. We uncovered evidence of genetic variation of epigenetic marks causally linked to diapause plasticity. Unfortunately, the sensitivity of diapause plasticity to genetic background precluded us from using RNAi to directly test the hypothesis that genotypes showing invariant histone abundance across reproductive states are insensitive to H3K4me3 and H3K36me1 manipulation. Most of the genetic background of our experimental genotypes comes from lab-derived, rather than wild, chromosomes (gray vs. blue, Fig 2B, see Methods). Future work screening additional histone marks may uncover distinct histone marks that vary in those lines that show invariant H3K4me3 and H3K36me3. Moreover, innovation in genome-wide histone mark profiling on minimal tissue, as well as epigenetic editing [86], will allow us to further probe the possibility that diapause in the ovary is regulated by distinct epigenetic mechanisms in distinct genotypes.

Genetic variation in the epigenetic determinants of diapause plasticity indicates that natural selection may act through chromatin to match phenotype and environment. Intriguingly, genes encoding many epigenetic factors are shaped by spatially-varying selection in *D*. *melanogaster*, including enzymes that methylate and demethylate H3K4 and H3K36 [87–89]. Future research comparing epigenetic regulation of diapause across a large sample of genotypes derived from the *D*. *melanogaster* latitudinal cline may offer the first glimpses of how evolution shapes the epigenetic mechanisms underlying adaptive variation in plasticity. Understanding these evolutionary processes is vital: genetic variation of epigenetic regulation likely shapes how natural populations respond to the extreme seasonal environments arising from ongoing climate change [90].

## Materials and methods

### *Drosophila* stock provenance, husbandry, and sequence diversity

We constructed the “PA” inbred line from an isofemale line collected from Media, Pennsylvania in July, 2018. To inbreed the line, we mated one brother and one sister each generation for 10 generations. We similarly constructed the genotypically distinct “FL” line from an isofemale line collected in Miami, Florida in July 2018. To assay the reduction in heterozygosity from 10 generations of inbreeding, we extracted DNA from pools of 50 individuals from each focal inbred line (PA and FL) and sequenced these pools at 100x coverage (Admera Health, South Plainfield, NJ). We trimmed raw reads using Trimmomatic (v.0.39) [91] and mapped reads to the *D*. *melanogaster* reference genome using BWA aligner (v.0.7.17)[92]. We used Popoolation [93], a software designed for analyzing pooled sequencing data, to calculate the per site heterozygosity (π). We found that the average per site heterozygosity is 0.0019 and 0.0025 in the PA and FL inbred lines, respectively. We used Popoolation2 [94] to calculate genetic divergence (F_st_) between the PA and FL inbred lines. Consistent with previous estimates of divergence between populations derived from these latitudes [95], we found that the PA-FL genome-wide F_st_ is 0.005. We deposited the PA and FL genome sequences on the NCBI Sequencing Read Archive under accession number PRJNA884433.

We maintained stocks at 25°C in 12-hour light/dark cycles on standard molasses food. The inbred and isofemale lines used in this study are available upon request. The provenance and diapause plasticity of the other lines used in the study (S6 Fig) can be found in S11 Table.

### Diapause assay

To assay diapause, we subjected 0–6 hour-old virgin females to 12°C and a short-day light cycle (9 hours of light, 15 hours of darkness) for 28 days. To assay whether a female was in diapause, we dissected out the ovaries and determined the latest developmental stage of the ovary as defined in Saunders *et al*. [1989, [45]]. Specifically, we designated females as undergoing diapause if they lacked vitellogenic egg chambers (stage 8 or later, Fig 1B). We designated females as “persistently reproductive” if both ovaries had one or more egg chambers at stage 8 or later. We excluded from experiments those rare females (<1%) whose ovaries fell between these categories.

To determine if laboratory simulation of diapause-inducing conditions recapitulated the differential survival of females that enter or fail to enter diapause at the start of winter [36,43,44], we counted the proportion of diapausing, persistently reproductive, and dead females held at 12°C for one, two, and three months. We found that the fraction of females in diapause remained constant across timepoints. In contrast, the fraction of reproductive individuals consistently decreased while the fraction of dead individuals consistently increased (S7 Fig). These data suggest that persistently reproductive individuals, but not diapausing individuals, are aging under our simulated winter conditions.

### Transgenerational assay

To assess transgenerational effects of diapause, we induced diapause as described above but determined the reproductive state of females without dissection using a modification of the “Bellymount” protocol described in [96]. We positioned females between two cover slips with a drop of 50% glycerol and determined the reproductive state by visualizing through the abdomen the presence or absence of egg chambers at stage 8 or later. To evaluate the accuracy of the Bellymount approach, we collected a pool of PA females subjected to simulated winter conditions and parsed this pool into diapause, persistently reproductive, and ambiguous, using the Bellymount. We found that 36 of 36 females designated (non-destructively) as diapausing indeed had ovaries that lacked egg chambers at stage 8 or later, revealed by dissection. Emboldened by this trial, we then crossed females in diapause (“naïve females”, Fig 1C and 1D) to males from the same inbred line at 25°C and then placed virgin female offspring from this cross either into a 12°C incubator (“daughters of diapause females,” Fig 1C and 1D) to assess diapause rate or into a vial with males at 25°C to generate granddaughters of the generation 0 diapause females. We repeated this process with these granddaughters as well as the great-granddaughters of the generation 0 diapause (“naïve”) females. We compared diapause plasticity of generation 0 to that of daughters, granddaughters, and great-granddaughters using a χ^2^ test. In parallel, we assayed the diapause plasticity of “naïve females” throughout the transgenerational assay (a total of six weeks). We found that the diapause plasticity in the first half of the experiment (72/80 individuals, 90%) and in the second half of the experiment (151/182 individuals, 83%) are statistically indistinguishable (χ^2^ = 2.170, p = 0.14). Furthermore, the diapause rates from each two-week period are significantly different from the diapause plasticity of daughters, granddaughters, and great-granddaughters from diapause (χ^2^>17.0,p<0.0001, S1 Table).

### RNA-sequencing and analysis

To define the gene expression associated with diapause while controlling for genotype, temperature, and age, we induced diapause as described above. We kept age-matched control flies as virgins in an incubator set to 25°C and a long-day light cycle (12 hours of light, 12 hours of dark) for 28 days. We flipped these control females onto fresh food every seven days due to accelerated mold growth on the food at 25°C. We isolated equivalent egg chamber stages from ovaries across reproductive states. For persistently reproductive ovaries and age-matched control ovaries, we dissected off accessory structures and then isolated by microdissection ovary stages 1–7 only (Fig 1B). For arrested ovaries, we removed the accessory structures only. We prepared total RNA (MirVana miRNA isolation kit, Thermo Fisher, Waltham, MA) from three replicates of 50 pooled ovaries for each condition in each genotype. In total, we prepared 18 samples (PA diapause, PA persistently reproductive, PA age-matched control, FL diapause, FL persistently reproductive, and FL age-matched control) and 18 libraries using NEBNext Ultra II (directional) with Poly-A selection, and sequenced libraries using Illumina 2x150 for a total of 30M reads per sample (Admera Health Biopharma Services, South Plainfield, NJ). All sequencing reads are available on the Sequencing Read Archive (NCBI), accession number PRJNA884433.

We trimmed raw reads using Trimmomatic (v.0.39) [91] and mapped reads to the *D*. *melanogaster* reference transcriptome using STAR aligner (v.2.7.10) [97]. We estimated expression levels using featureCounts [v.2.0.3,[98]], which assigned 95.3–95.9% of the reads to genes. We analyzed differential expression using DESeq2 (v.1.36.0) in R [99]. We discarded genes with fewer than 50 reads total across all samples in these analyses. We defined genes as significantly differentially expressed when the false discovery rate (FDR) was less than 0.05. Upon analyzing differential expression, we found that six and seven genes in the PA and FL lines, respectively, had an extremely high standard error (lfcSE >1) but were called as significantly differentially expressed between diapausing and persistently reproductive ovaries (FDR < 0.05). In both genotypes, only a single replicate of the (pooled) persistently reproductive ovaries had an elevated number of reads mapping to these genes. We discovered that these genes belong to the multi-copy chorion gene cluster, which undergoes selective, 15-80-fold gene amplification (endo-replication) in the ovarian follicle cells from ovary stages 8–14 [100]. This observation is consistent with a small amount of contamination of a later stage egg chamber. Indeed, the chorion genes accounted for the deviation of the single replicate from the other two replicates in both genotypes on the initial PCA plot. Consequently, we excluded these genes from our analyses and note that this exclusion had no effect on the conclusions drawn from the data. We note that our conclusions were insensitive to the featureCounts multimapping parameter choice (including the–M–fraction options).

To compare diapause-specific gene expression between the PA and FL lines, we used the age-matched control ovaries to subsample those genes that were differentially expressed between diapausing and persistently reproductive ovaries. We included in downstream analyses only those genes that were differentially expressed between diapausing and persistently reproductive ovaries *and* between diapausing and age-matched control ovaries in a given genotype. In other words, we excluded genes that are specifically up- or down-regulated in persistently reproductive ovaries compared to both diapause and age-matched controls. After generating this reduced list of significantly differentially expressed genes for both PA and FL genotypes, we compared across the two gene lists and determined which genes were differentially expressed in both PA and FL (“genotype-independent”), differentially expressed only in the PA line (“PA-specific”), or differentially expressed only in the FL line (“FL-specific”, see Fig 4A).

We performed pathway enrichment analysis of differentially expressed genes using the Reactome Pathway Database (v.81) [101]. We considered pathways significantly enriched if there were five or more genes in a given category and the FDR was less than 0.05.

All code implemented in these bioinformatic analyses can be found at https://github.com/LevineLabUPenn/Diapause-Genomics-2023.

### Western blotting and analysis

To assay histone mark abundance in the ovary, we isolated by microdissection ovary stages 1–7 (see above) in 1X PBS and ground the material in RIPA buffer (Cell Signaling Technology, Danvers, MA), Protease Inhibitor Cocktail (Roche, Basel, Switzerland), and PMSF (Cell Signaling Technology, Danvers, MA). To promote solubility, we incubated the lysate in Benzonase (Sigma Aldrich, St. Louis, MO) for 30 min at 4°C. We probed the blots with anti-H3K4me3 (Active Motif, Carlsbad, CA), anti-H3K36me1 (Abcam, Cambridge, UK), anti-H3K27me3 (Active Motif, Carlsbad, CA), anti-H3K27ac (Abcam, Cambridge, UK), anti-H3K9me3 (Abcam, Cambridge, UK), and anti-H3K9ac (Abcam, Cambridge, UK) at 1:1000 dilution. We probed with anti-α-tubulin (Developmental Studies Hybridoma Bank, Iowa City, IA) as a loading control (also 1:1000 dilution). We used anti-mouse and anti-rabbit HRP secondaries (Kindle Biosciences, Greenwich, CT) both at 1:1000. We exposed the blots with Kwikquant western blot detection kit and imaged with a Kwikquant imager (Kindle 277 Biosciences, Greenwich, CT). We quantified relative fluorescence of marks according to Stael *et al*. (*2022)* and normalized all measurements to diapause [102]. We ran a third biological replicate only if we detected consistent differences in abundance across two replicates. For marks H3K4me3 and H3K36me1, we ran three biological replicates and compared abundance across diapausing and reproductive ovaries using a Mann-Whitney U test.

### Tissue-specific knockdown of histone writers and erasers and analysis of diapause plasticity

To knockdown expression of histone mark writers and erasers, we took advantage of preconstructed D. melanogaster lines from the Transgenic RNAi Project [103]. These lines encode “Upstream Activating Sequence” (UASp)-driven short hairpins (shRNA) that target transcripts encoding *D*. *melanogaster JHDM2* (Bloomington Drosophila Stock Center “BDSC” #32975), Set2 (BDSC #55221), Set1 (BDSC #33704), and lid (BDSC #36652). These cassettes are inserted into attP landing sites. Given the well-known effects of genetic background on diapause plasticity [46,48,68,69], we carefully introgressed the chromosome encoding each UASp-shRNA construct (*Set2* and *lid* on chromosome II, *JHDM2* and *Set1* on chromosome III) into the PA inbred line (see S8 Fig for crossing scheme). Furthermore, we ensured no recombination using a combination of balancer chromosomes and transmission only through males (which do not recombine) to tightly control the genetic background of the control and experimental flies (S8 Fig). Moreover, we only compared experimental genotypes encoding the UASp-shRNA construct in a given attP site to control genotypes, which encode the same chromosome with the same attP site but lacking the UASp-shRNA construct (BDSC #36304 for chromosome II, BDSC #36303 for chromosome III). To verify the presence of these constructs after the multi-generation crossing scheme, we used PCR to amplify the AmpR gene introduced along with the UAS-shRNA construct (S13 Table). We crossed these stable stocks to the MTD-Gal4 driver (BDSC #31777), which expresses the GAL4 transcription factor throughout the ovary [104].

To validate the knockdown of transcription by RNAi, we performed RT-qPCR on RNA prepared from ovaries stages 1–7 in control and experimental shRNA genotypes at 48 hours after eclosion at 25°C (S13 Table). To validate the depletion or enrichment of histone mark abundance by RNAi against the target chromatin writers and erasers, we conducted western blotting (as above) on protein lysate prepared from stages 1–7 ovaries from control and experimental shRNA genotypes 48 hours after eclosion at 25°C. We note that RNAi efficiency decreases with decreasing temperature [105,106], disabling us from distinguishing between the effects of RNAi on diapause entry and maintenance. We also note that these enzyme manipulations may deplete or enrich multiple marks. For example, *Set2* knockdown may decrease H3K36me2 and H3K36me3 in addition to H3K36me1. Similarly, knocking down *Set1* and *lid* may also alter the abundance of H3K4me2 in addition to H3K4me3. Given that mono- di- and tri-methylation of the same histone lysine are generally found in different regions of the same genes [107,108], it is unlikely that these related marks have distinct impacts on diapause plasticity.

To determine whether experimental manipulation of histone mark abundance altered diapause plasticity, we assayed diapause in control and experimental RNAi lines by dissecting ovaries after 28 days in diapause-inducing conditions, as described above. We analyzed diapause plasticity using an odds ratio (implemented in R) that compares diapause plasticity between control and RNAi lines. An odds ratio represents the change in likelihood of diapause given the presence of transcript knockdown. To ensure that activation of the RNAi machinery alone does not affect diapause plasticity [e.g.,[109]], we introduced a chromosome carrying UASp-mCherry into the PA inbred line (BDSC #35785) and assayed diapause plasticity after 28 days under simulated winter conditions (see above). We found that expressing hairpins cognate to an absent target did not significantly affect diapause plasticity (no-UAS control plasticity = 63.4%, n = 101, mCherry RNAi plasticity = 71.4%, n = 98, odds ratio = 1.45, p>0.05)

*D*. *melanogaster* ovary development depends in part on chromatin-mediated gene regulation. We sought to rule out the possibility that the observed increase in incidence of arrest (“diapause plasticity”) upon transcript knockdown was simply due to a block in ovary development, independent of diapause. This was of particular concern given that experimental depletion of H3K36me1 and H3K4me3 increased diapause to nearly 100%. We reasoned that increasing the dynamic range of diapause plasticity in both experimental and control lines could allow us to determine if ovary development was blocked upon transcript knockdown at 12°C. To decrease the baseline diapause plasticity in both experimental and control lines, we took advantage of the transgenerational decrease in diapause plasticity (Fig 1D). We exposed females from the UAS-shRNA and control lines to diapause conditions and assayed ovary development using the modified Bellymount method described above. We then crossed females in diapause to the MTD-Gal4 driver (BDSC #31777), and exposed daughters from this cross to simulated winter conditions and assayed for diapause plasticity. We found that fewer than 30% of these daughters have arrested ovaries, suggesting that *Set1* or *Set2* knockdown alone does not block ovary development at 12*°C* (S3B Fig).

To assay the effect of genetic background on H3K4me3- and H3K36me1-mediated diapause plasticity, we introduced chromosomes encoding either the UASp-shRNA construct, or the control chromosomes lacking the UASp-shRNA, into the FL inbred line. We found that, unfortunately, the three GAL4- and the single UAS- encoding control chromosomes, rather than the two FL control chromosomes, determined the diapause plasticity of our experimental strains. Specifically, the diapause rates of the FL control lines were similar to those of the PA control lines (Chr2: 92.9%, Chr3: 58.3%, compare to Fig 2C). Consequently, we were unable to test directly the effect of genetic background on the capacity of H3K4me and H3K36me to alter diapause plasticity.

### Immunofluorescence and image analysis

To evaluate whether the FL line entered canonical diapause like the temperate PA line, we conducted immunofluorescence on ovaries following the protocol described in [110]. To assay germline stem cell number, we stained ovaries with anti-α-spectrin (1:300, Developmental Studies Hybridoma Bank, Iowa City, IA) and anti-Vasa (1:50, Developmental Studies Hybridoma Bank, Iowa City, IA). Following criteria described in [111], we defined germline stem cells as the anterior-most Vasa-positive cells in the stem cell niche that display an anterior α-spectrin signal (the “spectrosome”). Scanning through each z-stack, we counted the number of germline stem cells from 10 germaria in 5 ovary pairs, for a total of 50 germaria. We used a 2-way ANOVA to evaluate the statistical significance of the effects of treatment (diapause and age-matched control) and time (28 days in treatment vs. 5 days recovered from treatment). To assay double-strand break abundance, we stained ovaries with anti-γ-H2Av (1:1000, Developmental Studies Hybridoma Bank, Iowa City, IA). To quantify the average fluorescence of γ-H2Av in ovaries, we outlined representative stage 4 egg chambers with the Freehand tool in FIJI (v.1.0) [112]. Also in FIJI, we calculated the fluorescent signal intensity using the polygon tool to define the borders of the tissue. We used the “measure tool” to calculate the mean pixels within these boundaries. We normalized all fluorescence intensity values of the PA line to the mean intensity value of the age-matched control in PA, and all fluorescence intensity values of the FL line to the mean intensity value of the age-matched control in FL. We calculated fluorescence from 10 replicates of stage four egg chambers in five ovary pairs, for a total of 50 egg chambers. We compared the mean fluorescence of γ-H2av in diapause and age-matched control ovaries using a Mann-Whitney U test (implemented in R).

For all immunofluorescence experiments, we mounted ovaries with ProLong Gold Antifade Reagent with DAPI (Thermo Fisher Scientific, Waltham, MA). We imaged slides at 63x magnification on a Leica TCS SP8 Four Channel Spectral Confocal System. For each experiment, we used the same imaging parameters across genotypes and reproductive state.

### Fertility assay

To further evaluate whether the FL line entered canonical diapause similarly to the temperate PA line, we assayed diapause-induced preservation of fertility in both lines (S4 Fig). We counted progeny of females crossed to wildtype (w^1118^) males after diapause exit at 28 days in 12°C. We compared these females to age-matched controls (maintained at 25°C for 28 days) crossed in parallel to wildtype (w^1118^) males. In each vial, we crossed three females and six males. We replicated each cross across 12 vials and flipped each cross onto fresh food every three days. To exclude age-dependent male fertility effects, we replaced the six males every three days with one-to-three-day old males. We recorded the number of adult progeny from each flip. We compared the mean number of progeny from diapause and age-matched control using a Mann-Whitney U test (implemented in R).

## Supporting information

S1 Table Diapause plasticity of naïve females (at two timepoints) and of the daughters, granddaughters, and great-granddaughters reported in Fig 1D. (XLSX)

S2 Table Results of the DESeq analysis of differential gene expression between diapause and age-matched control ovaries of the PA inbred line reported in S1 Fig. (XLSX)

S3 Table Results of the DESeq analysis of differential gene expression between diapause and reproductive ovaries of the PA inbred line, reported in Figs 1G and S1B. (XLSX)

S4 Table Results of histone mark quantification of western blots reported in Figs 2A and S2. (XLSX)

S5 Table Results of germline stem cell number quantification reported in Fig 3B. (XLSX)

S6 Table Results of quantification of y-H2av signal intensity reported in Fig 3C. (XLSX)

S7 Table Total progeny number from diapaused and age-matched control females reported in S4 Fig. (XLSX)

S8 Table Results of the DESeq analysis of differential gene expression between diapause and persistently reproductive ovaries of the FL inbred line reported in S5D Fig. (XLSX)

S9 Table Results of the DESeq analysis of differential gene expression between diapause and age-matched control ovaries of the FL inbred line reported in S5C Fig. (XLSX)

S10 Table Fold change comparison of gene expression between diapause and reproductive ovaries in PA and FL inbred lines reported in Fig 4A. (XLSX)

S11 Table Provenance and plasticity of *Drosophila* lines used in this study. (XLSX)

S12 Table Results of histone mark quantification of western blots reported in S6 Fig. (XLSX)

S13 Table Primers used in this study. (XLSX)

S1 Fig Principal component analysis (PCA) of RNA-seq reads from the temperate North American inbred line. (A) PCA of RNA-seq reads from diapausing (square), persistently reproductive (circle), and age-matched control (triangle) ovaries, stages 1–7 only. Note that temperature explains most of the variance between the three samples (PC1, 90%). (B) PCA of RNA-seq reads from diapausing and persistently reproductive ovaries only.
(TIF)

S2 Fig Western blots probed for various histone marks on multiple biological replicates. Blots of ovary lysate prepared from diapausing and persistently reproductive ovaries and quantification relative to α-tubulin loading control of (A) H3K4me3, (B) H3K36me1, (C) H3K27ac, (D) H3K9ac, (E) H3K27me3, and (F) H3K9me3. Blue boxes delineate replicates shown in Fig 2A, yellow boxes delineate replicates shown in Fig 4B. D = diapause, PR = persistently reproductive. PA = focal Pennsylvania-derived inbred line, FL = focal Florida-derived inbred line.
(TIF)

S3 Fig Quality control of RNAi experiments. (A) RT-qPCR confirming knockdown (KD) of transcripts targeted by RNAi. Note *Set2* and *lid* knockdown genotypes are compared to chromosome II control genotype, while *Set1* and *JHDM2* knockdown genotypes are compared to chromosome III control genotype. (B) Number of diapause and persistently reproductive females in winter-simulated conditions upon histone mark writer knockdown (*Set1* or *Set2*) in the ovaries of females whose mothers had not undergone diapause (pink, data from Fig 2C), or who had undergone diapause (blue, “transgenerational”). The abundance of persistently reproductive ovaries in both genotypes under the transgenerational treatment verified that knockdown of *Set1* or *Set2* alone does not block ovary development at 12°C (see Methods). “chr.” = chromosome.
(TIF)

S4 Fig Total progeny from diapause and age-matched control females of PA (blue) and FL line (yellow). Each replicate represents a vial of three females. t-test, *** p<0.001, ** p<0.01. n = 12 replicate vials.
(TIF)

S5 Fig Heat map and principal component analysis (PCA) of FL line RNA-seq reads. (A) Heatmap of the top 200 significantly differentially expressed genes (by FDR) between age-matched control, diapausing, and persistently reproductive ovaries, stages 1–7 only. Blue-red gradient depicts the Z-score of each gene. Red corresponds to upregulated genes and blue corresponds to downregulated genes. (B) Heatmap of the top 200 significantly differentially expressed genes (by FDR) between diapausing and persistently reproductive ovaries only. Blue-red gradient depicts the Z-score of each gene. Red corresponds to upregulated genes and blue corresponds to downregulated genes. (C) PCA of RNA-seq reads from diapausing (square), persistently reproductive (circle), and age-matched control (triangle) ovaries. Note that temperature explains most of the variance among the three samples (PC1, 87%). (D) PCA of RNA-seq reads from diapausing and persistently reproductive ovaries.
(TIF)

S6 Fig H3K4me3 and H3K36me1 abundance across replicate lines. Blots of ovary lysate prepared from diapausing and persistently reproductive ovaries and quantification relative to α-tubulin loading control of H3K4me3 and H3K36me1 in (A) PA-2, (B) FL-2, (C) PA-3, (D) PA-4, (E) PA-5, and (F) FL-3 genotypes. D = diapause, PR = persistently reproductive. t-test, ** p<0.01, * p<0.05, n.s, p>0.05. See S11 Table for descriptions of genotypes.
(TIF)

S7 Fig Simulated winter conditions recapitulate differential survival of diapausing and persistently reproductive females. Results from assay of female state under simulated winter conditions for one, two, or three months. “n” corresponds to the sample size of females assayed at a given timepoint.
(TIF)

S8 Fig Crossing scheme used to generate RNAi and control lines. Brown dashed boxes correspond to lines constructed from cross A (bottom) and green dashed boxes correspond to lines constructed from cross B (bottom).
(TIF)
