# Supplementary figures and images for: Histone methylation regulates reproductive diapause in Drosophila melanogaster
Source: PLoS Genet. 2023 Sep 13;19(9):e1010906. doi: 10.1371/journal.pgen.1010906 (PMC10499233; doi:10.1371/journal.pgen.1010906)

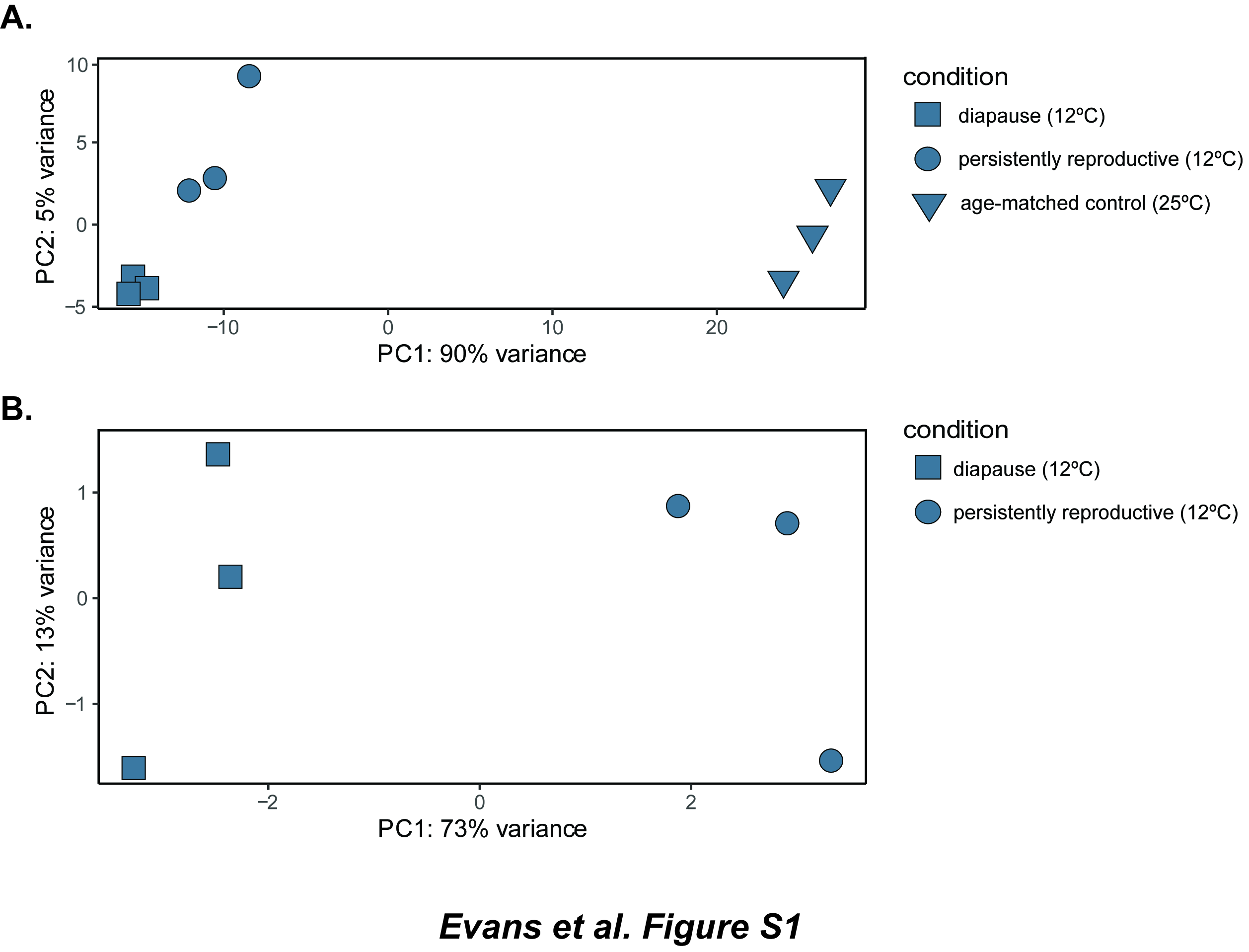

Supplement: S1 Fig — (A) PCA of RNA-seq reads from diapausing (square), persistently reproductive (circle), and age-matched control (triangle) ovaries, stages 1–7 only. Note that temperature explains most of the variance between the three samples (PC1, 90%). (B) PCA of RNA-seq reads from diapausing and persistently reproductive ovaries only. (TIF) [file pgen.1010906.s014.tif]

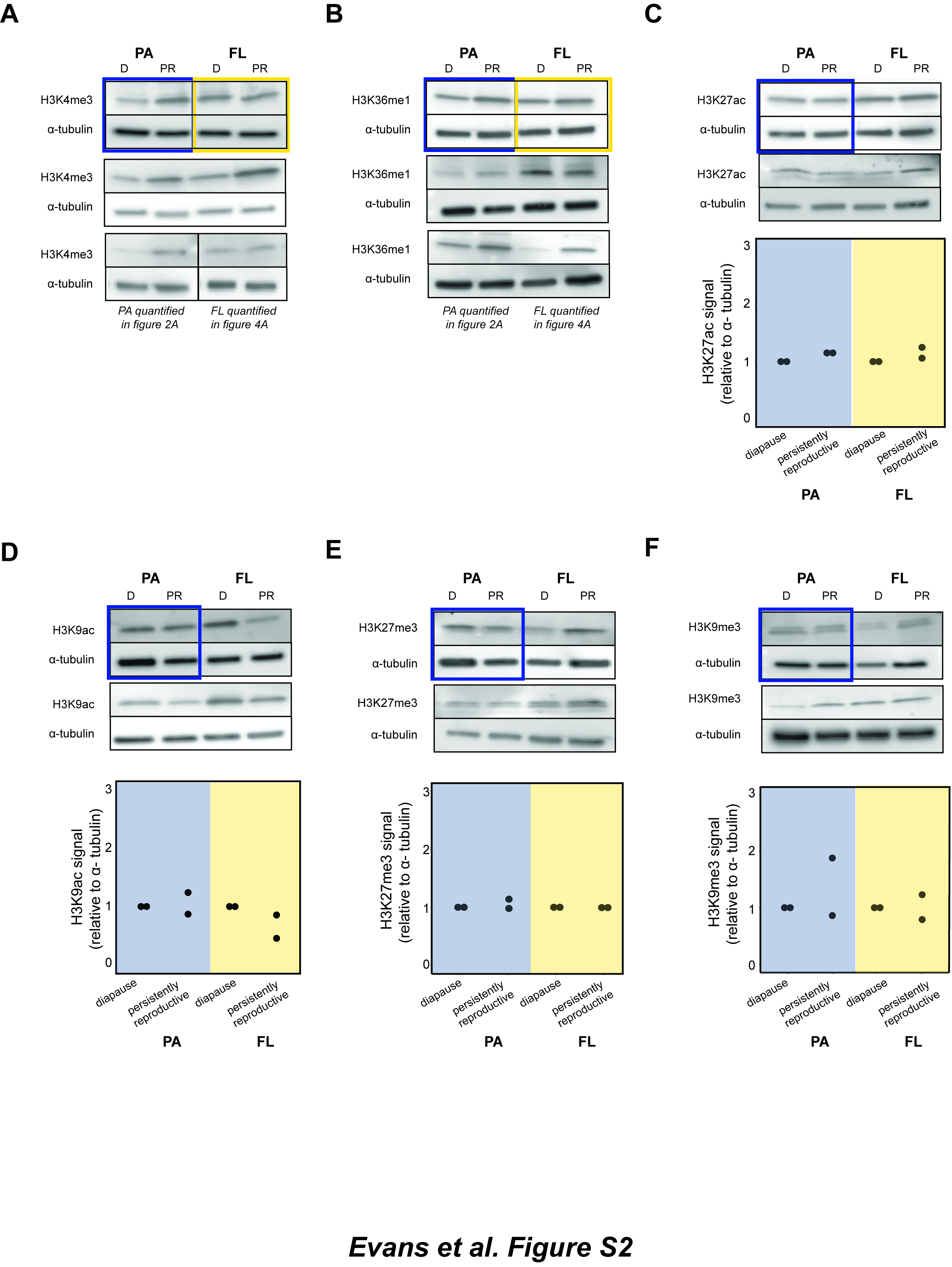

Supplement: S2 Fig — Blots of ovary lysate prepared from diapausing and persistently reproductive ovaries and quantification relative to α-tubulin loading control of (A) H3K4me3, (B) H3K36me1, (C) H3K27ac, (D) H3K9ac, (E) H3K27me3, and (F) H3K9me3. Blue boxes delineate replicates shown in Fig 2A, yellow boxes delineate replicates shown in Fig 4B. D = diapause, PR = persistently reproductive. PA = focal Pennsylvania-derived inbred line, FL = focal Florida-derived inbred line. (TIF) [file pgen.1010906.s015.tif]

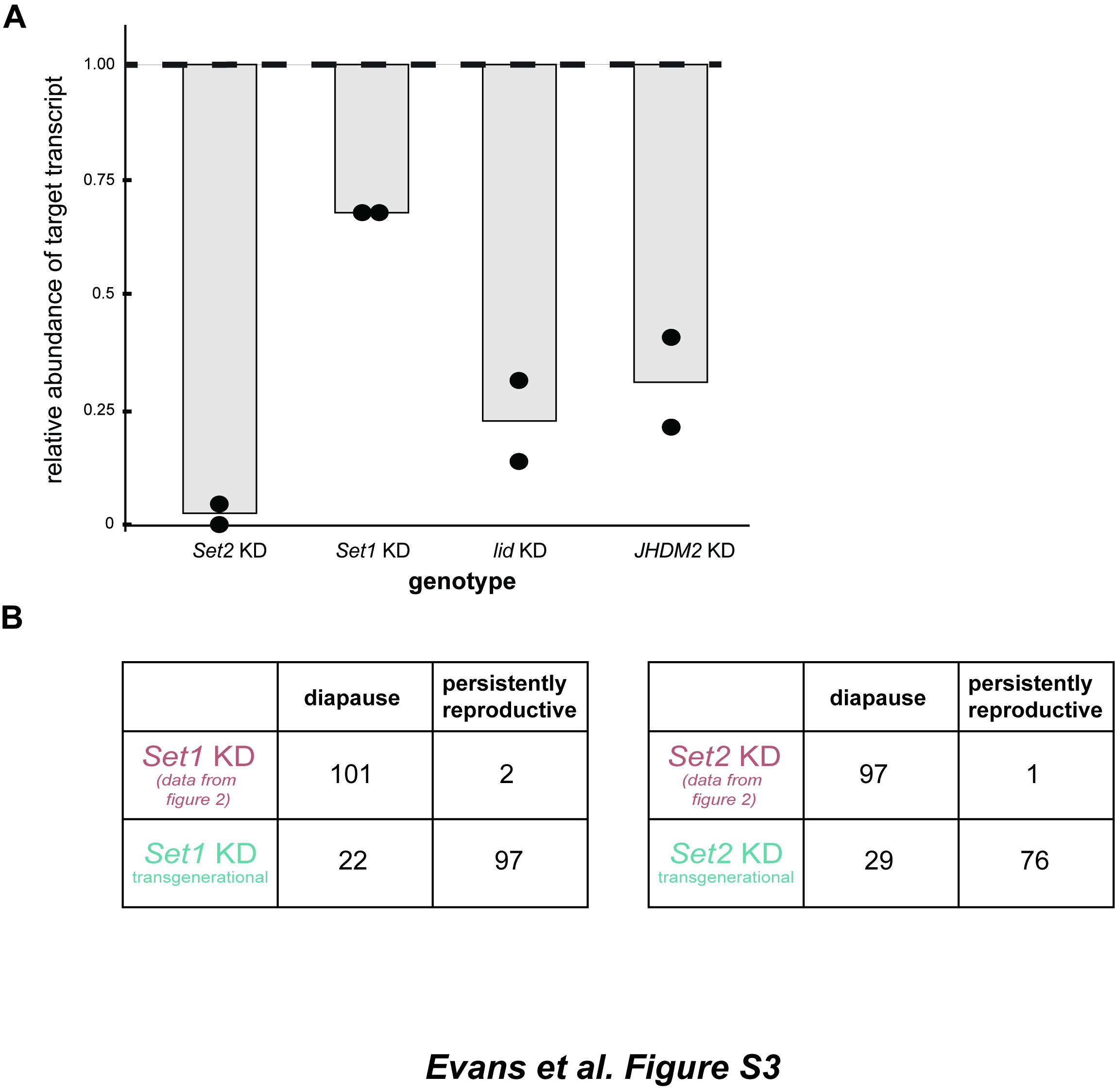

Supplement: S3 Fig — (A) RT-qPCR confirming knockdown (KD) of transcripts targeted by RNAi. Note Set2 and lid knockdown genotypes are compared to chromosome II control genotype, while Set1 and JHDM2 knockdown genotypes are compared to chromosome III control genotype. (B) Number of diapause and persistently reproductive females in winter-simulated conditions upon histone mark writer knockdown (Set1 or Set2) in the ovaries of females whose mothers had not undergone diapause (pink, data from Fig 2C), or who had undergone diapause (blue, “transgenerational”). The abundance of persistently reproductive ovaries in both genotypes under the transgenerational treatment verified that knockdown of Set1 or Set2 alone does not block ovary development at 12°C (see Methods). “chr.” = chromosome. (TIF) [file pgen.1010906.s016.tif]

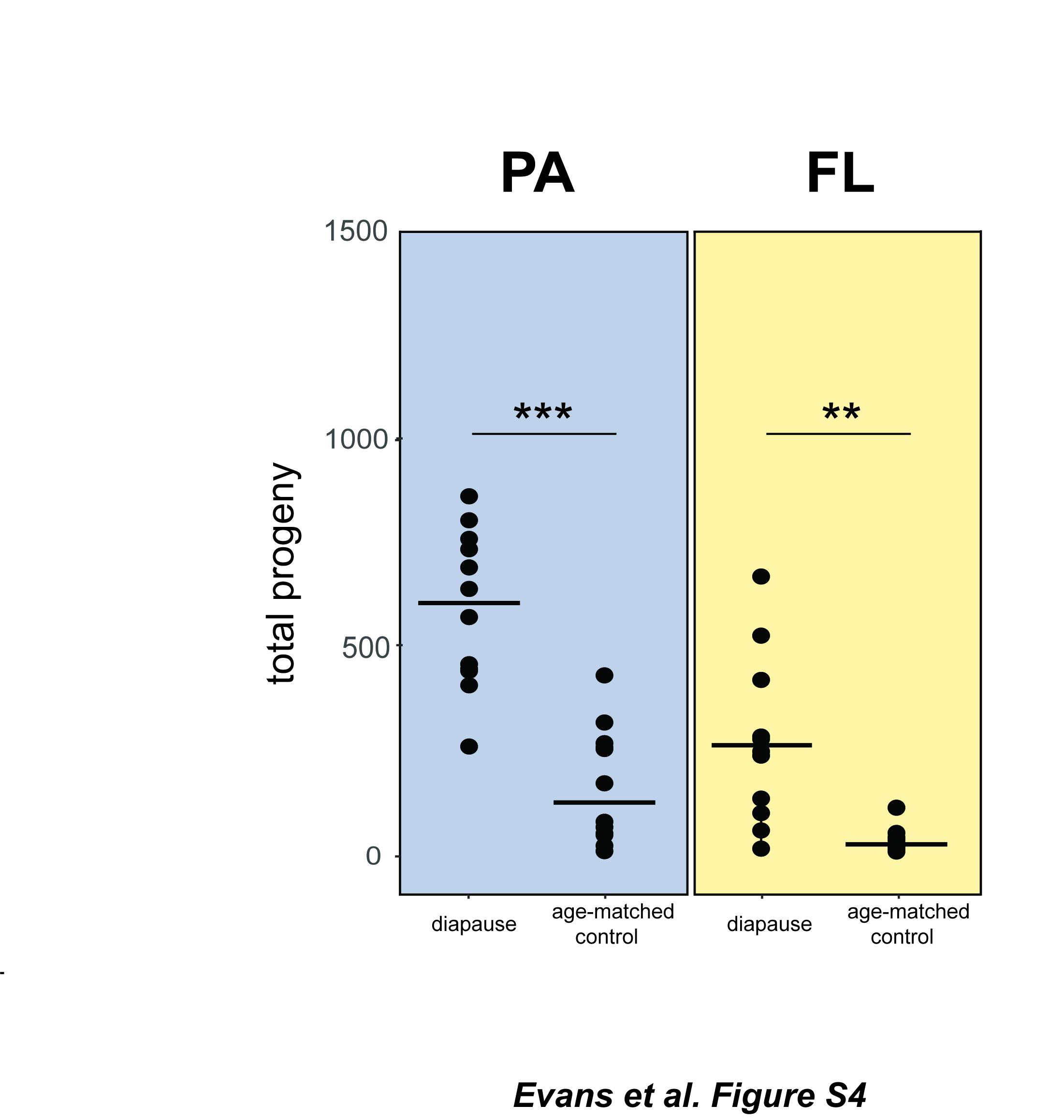

Supplement: S4 Fig — Each replicate represents a vial of three females. t-test, *** p<0.001, ** p<0.01. n = 12 replicate vials. (TIF) [file pgen.1010906.s017.tif]

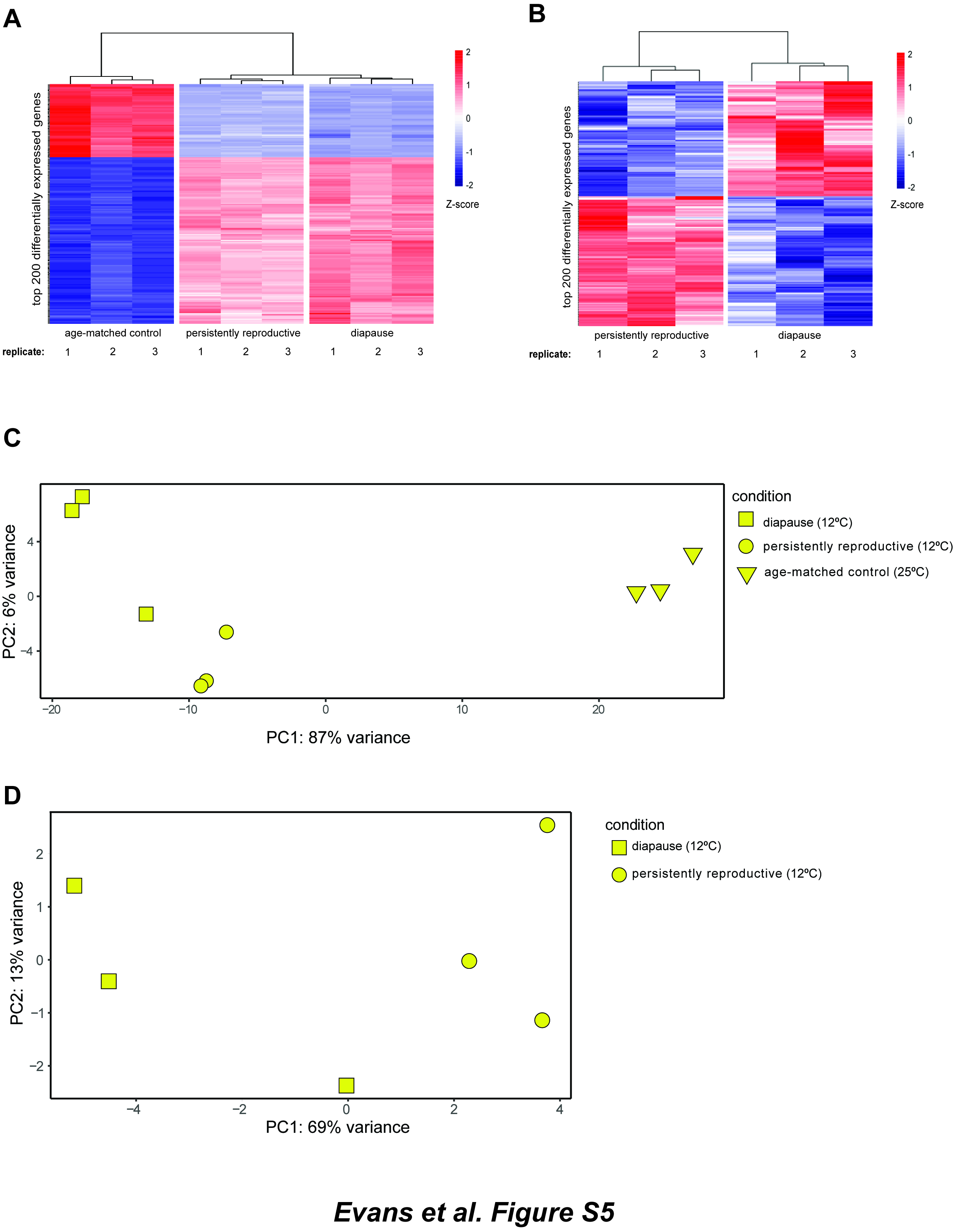

Supplement: S5 Fig — (A) Heatmap of the top 200 significantly differentially expressed genes (by FDR) between age-matched control, diapausing, and persistently reproductive ovaries, stages 1–7 only. Blue-red gradient depicts the Z-score of each gene. Red corresponds to upregulated genes and blue corresponds to downregulated genes. (B) Heatmap of the top 200 significantly differentially expressed genes (by FDR) between diapausing and persistently reproductive ovaries only. Blue-red gradient depicts the Z-score of each gene. Red corresponds to upregulated genes and blue corresponds to downregulated genes. (C) PCA of RNA-seq reads from diapausing (square), persistently reproductive (circle), and age-matched control (triangle) ovaries. Note that temperature explains most of the variance among the three samples (PC1, 87%). (D) PCA of RNA-seq reads from diapausing and persistently reproductive ovaries. (TIF) [file pgen.1010906.s018.tif]

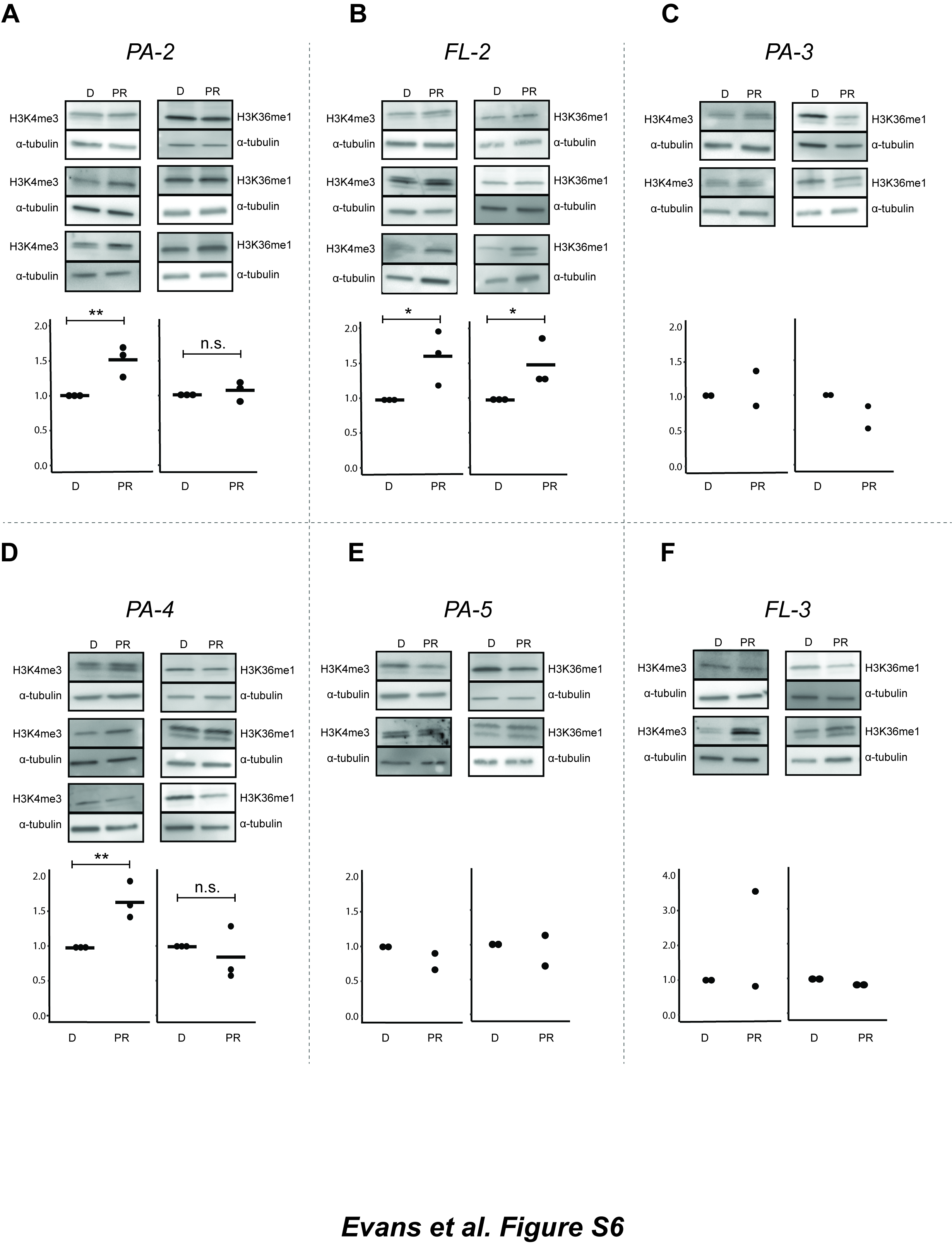

Supplement: S6 Fig — Blots of ovary lysate prepared from diapausing and persistently reproductive ovaries and quantification relative to α-tubulin loading control of H3K4me3 and H3K36me1 in (A) PA-2, (B) FL-2, (C) PA-3, (D) PA-4, (E) PA-5, and (F) FL-3 genotypes. D = diapause, PR = persistently reproductive. t-test, ** p<0.01, * p<0.05, n.s, p>0.05. See S11 Table for descriptions of genotypes. (TIF) [file pgen.1010906.s019.tif]

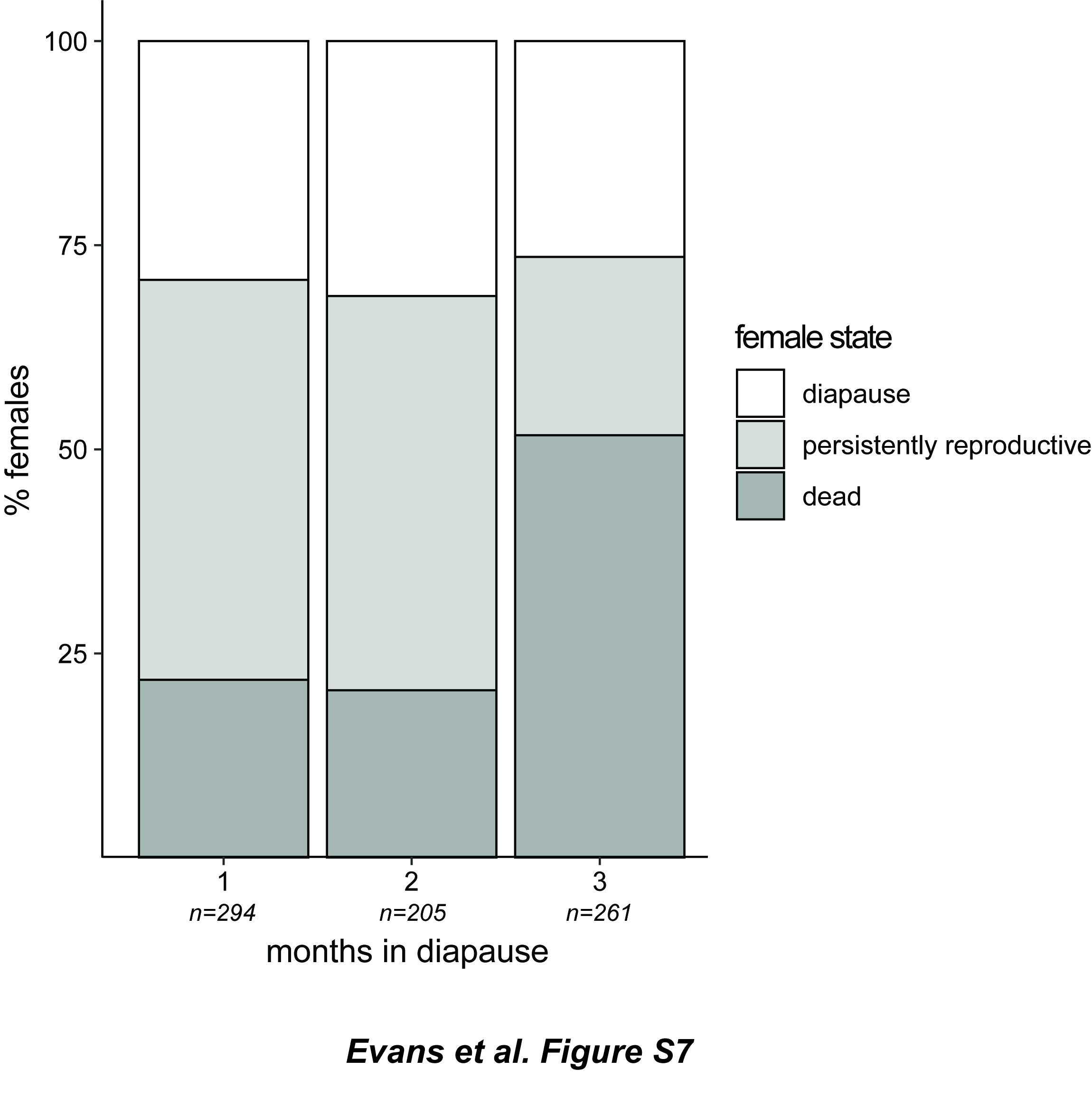

Supplement: S7 Fig — Results from assay of female state under simulated winter conditions for one, two, or three months. “n” corresponds to the sample size of females assayed at a given timepoint. (TIF) [file pgen.1010906.s020.tif]

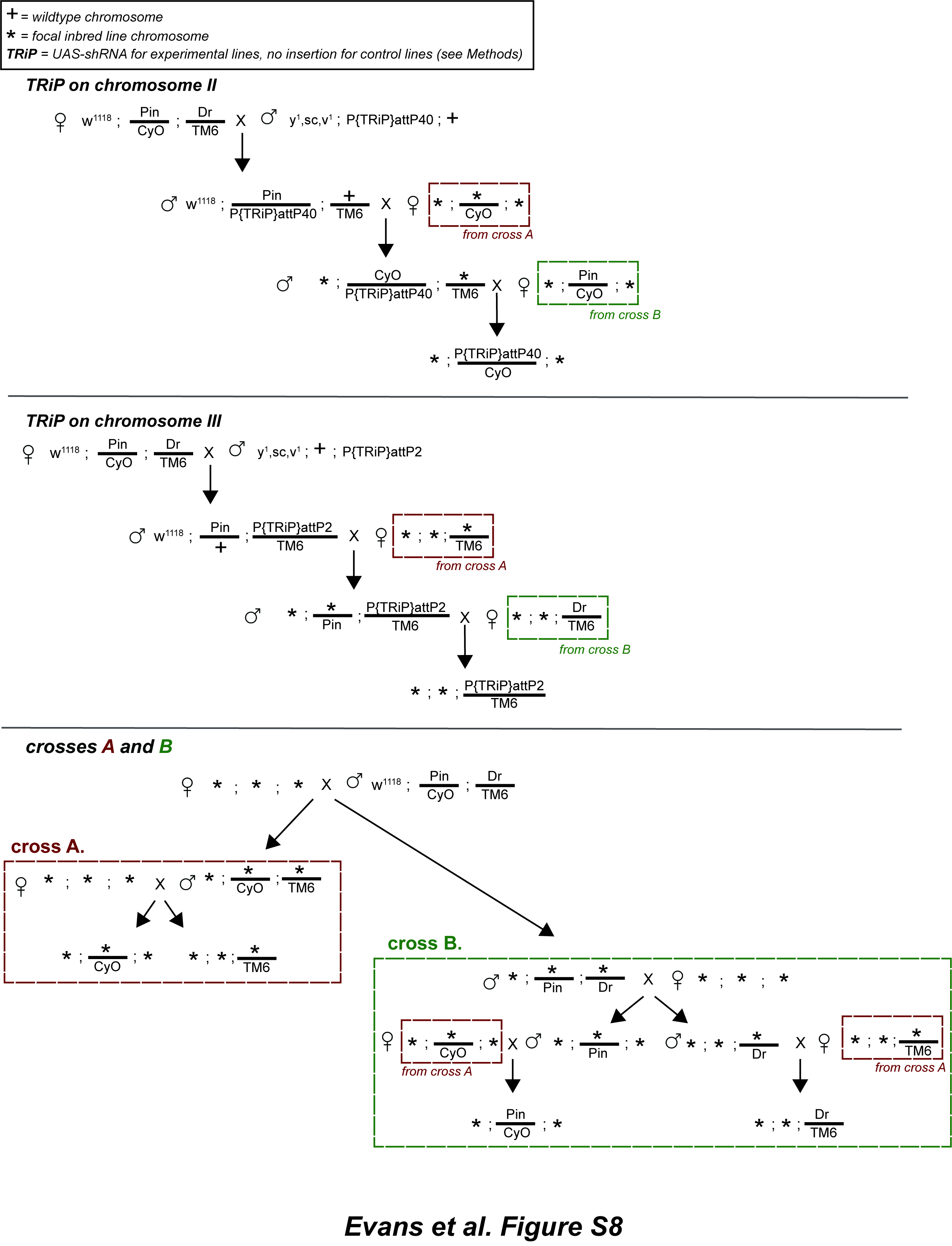

Supplement: S8 Fig — Brown dashed boxes correspond to lines constructed from cross A (bottom) and green dashed boxes correspond to lines constructed from cross B (bottom). (TIF) [file pgen.1010906.s021.tif]
